# The epidemiology and clinical presentation of acute vestibular syndromes: a systematic review of the literature

**DOI:** 10.1007/s00415-026-13720-5

**Published:** 2026-03-02

**Authors:** Flavio A. Coban, Alexander A. Tarnutzer

**Affiliations:** 1 Faculty of Medicine, University of Zurich, Zurich, Switzerland; 2 School of Medicine, University of St., Gallen, Switzerland; 3 Neurology, Cantonal Hospital of Baden, 5404 Baden, Switzerland

**Keywords:** Vestibular, Acute ischemic stroke, Epidemiology, Vertigo, Dizziness, Systematic review, Emergency department

## Abstract

**Background:**

Acute vertigo and dizziness are among the most frequent complaints presenting to the emergency department (ED). If acute-onset and persistent vertigo/dizziness (lasting > 24 h) are accompanied by motion intolerance, gait imbalance, nausea/vomitus, and (usually also) nystagmus, this refers to the acute vestibular syndrome (AVS). We aimed at collecting epidemiologic data about the frequency of AVS etiologies for improving the diagnostic workup.

**Methods:**

We conducted a Prospero-registered systematic literature search (MEDLINE, Embase – in June 2024) retrieving information on AVS’s clinical presentation, differential diagnosis, and stroke frequency. Two independent reviewers screened abstracts and full texts. Studies were rated with QUADAS-2 for risk of bias. Due to heterogeneity, we refrained from meta-analysis.

**Results:**

We identified 6731 unique articles and 45 manuscripts (reporting on > 5 Mio. dizzy patients) that were included. 3.45% of all ED visits were related to dizziness/vertigo. One tenth (i.e., 0.3%) met the diagnostic criteria for an AVS. Only about 74% of AVS patients received peripheral or central vestibular diagnoses, while 26% were non-specific. Stroke was the second most common cause (21% in our dedicated review) after acute unilateral vestibulopathy (38%). Selected studies assessing infarction location showed posterior-inferior cerebellar artery (36%) involvement most commonly. Stroke cases were seen at similar rates in patients with or without nystagmus (22 vs. 25%).

**Conclusion:**

Representing 10% of all dizzy patients, AVS in the ED is frequent. With a stroke fraction among all AVS cases of approximately 21%, profound clinical training to differentiate peripheral from central causes reliably should be prioritized.

**Supplementary Information:**

The online version contains supplementary material available at https://doi.org/10.1007/s00415-026-13720-5.

## Introduction

Acute vertigo and dizziness are among the most frequent presenting symptoms in the emergency department (ED). In a subset of patients, reported symptoms will be prolonged, meeting diagnostic criteria for an acute vestibular syndrome (AVS). The AVS is defined as a “clinical syndrome of acute-onset, continuous vertigo, dizziness, or unsteadiness lasting days to weeks, and generally including features suggestive of new, ongoing vestibular system dysfunction (e.g., vomiting, nystagmus, postural instability)” [1]. Underlying causes of AVS spread across specialties and range from benign self-limiting conditions to life-threatening diseases. Thus, in the ED, the highest priority is to identify those patients with dangerous central causes requiring immediate treatment.

In recent decades, advancements in the diagnostic approach to the acutely dizzy patient, including those meeting diagnostic criteria for AVS, have been made. This includes focusing on subtle ocular motor signs by implementing the HINTS (Head Impulse, Nystagmus, Test of Skew) algorithm, which has been shown to be more sensitive than an early (i.e., obtained within the first 24–48 h) brain MRI (magnetic resonance imaging) including diffusion-weighted imaging (DWI) in detecting a vertebrobasilar stroke [2, 3].

Those modalities aim to exclude a stroke as a potential cause of AVS and generally differentiate between central AVS (cAVS) and peripheral AVS (pAVS). While diagnostic accuracy studies have advanced the field, systematic data on the epidemiologic distribution of specific diagnoses among AVS patients remain fragmented.

We conducted a systematic review to address epidemiologic features of acute vertigo focusing on AVS in the ED. Following PRISMA guidelines, we aimed to quantify (1) the prevalence of dizziness and AVS among emergency presentations, (2) the proportion of strokes across clearly defined AVS subgroups, and (3) the distribution of specific peripheral, central, and non-specific diagnoses, including episodic vestibular syndromes misclassified as AVS.

## Methods

### Search strategy

This review was prospectively registered in the PROSPERO database (ID: CRD42024518735 [4]). The search strategy was designed by a neurologist with relevant domain expertise in neuro-otology (AAT). We searched MEDLINE and Embase on the 19.06.2024 for English language articles, with our strategy focusing on the following components: (1) vertigo, dizziness, or gait imbalance, (2) epidemiology, clinical presentation, and differential diagnosis, (3) acute vestibular syndrome (including ischemic stroke and acute unilateral vestibulopathy) and (4) acute presentation. We also performed a manual search of reference lists from eligible articles and contacted corresponding authors where necessary. We did not seek to identify research abstracts from meeting proceedings or unpublished studies.

### Inclusion and exclusion rules for abstracts and full-text manuscripts

All gathered literatures were subject to title/abstract screening by two independent reviewers (AAT/FAC). Abstract review coding rules are provided in Appendix 1. Full-text screening was applied to all citations considered eligible or possibly eligible by at least one reviewer. Two independent reviewers (AAT/FAC) determined whether full-text manuscripts are eligible and, if not, provided a reason for exclusion (see Appendix 1). Differences were resolved by discussion and consensus. Inter-rater agreement on full-text inclusion was calculated using Cohen’s kappa [5]. No automation tools were used during the process.

### Data extraction

Data extraction of full-text eligible articles was done by one reviewer (FAC) using Systematic Review Data Repository Plus (SRDR + , [6]) by the U.S. governmental Agency for Healthcare Research and Quality (AHRC), a free tool for organizing and collaborating on systematic reviews.

Every study was analyzed for the inclusion of our sought outcomes (Proportion of dizziness in the ED, proportion of AVS among dizziness presentations, stroke incidence, and stroke territories) within a specific item-based data extraction form. Other predefined extraction items (variables) were study name, authors, journal, year of publication, study design, study type (including whether recruitment was consecutive or not), number and location(s) of research center(s), time frame, affiliation with a larger trial, patient inclusion and exclusion criteria, patient assessment setting (including the department e.g., emergency department (ED), level of training/specialization of the assessing physician), assessment batteries (with a focus on HINTS), reference test to confirm the diagnosis or other imaging modalities, and time from physician assessment to imaging and were applied over all reports included.

Published patient diagnoses were collected and grouped, including additional information such as time from symptom onset to presentation, time from hospital arrival to imaging, patients’ characteristics (such as age and gender, prodromal symptoms, and type of dizziness symptoms) and AVS definitions. When any variable was not explicitly described, they were coded as ‘not reported’ or NR (= not reported) and no assumptions were made.

With a false-negative rate of early (i.e., within the first 24–48 h) brain MRI-DWI, about 20% for detecting posterior circulation strokes as the underlying cause of an acutely dizzy patient [7] on false-negative MRIs was also abstracted (if available).

Our date extraction protocol was first piloted over ten studies, subsequently refined and then applied over all included articles. The abstracted data were then exported and analyzed using Microsoft Excel (Microsoft Corporation, Redmond, WA, USA).

## Data availability statement

Full data access was available through SRDR + (SRDR + stopped operations in 11/2025) and is now available upon reasonable request. Supplementary material includes literature search (Appendix 1), QUADAS2 ratings (Appendix 2), and the study characteristics (Appendix 3). Further inquiries can be directed to the corresponding author.

### Risk of bias assessment

All included studies underwent a structured risk of bias (RoB) assessment. Both reviewers (AAT/FAC) independently assessed the RoB and managed conflicts through consensus. An adapted version of Quality Assessment of Diagnostic Accuracy Studies included in Systematic Reviews 2 (QUADAS2) [4] was used due to the high occurrence of diagnostic accuracy studies and the predefined abstraction of performed clinical tests on AVS patients. The applied risk of bias assessment includes 15 items (formulated as questions) mainly answerable through “high risk”, “low risk”, or “unclear” and was done through SRDR + and exported to Excel. The complete RoB profile is provided in Appendices 2 and 3.

### Methods for synthesis

For a given research question, eligible studies were searched in our Excel database. Study eligibility was determined by searching key study characteristics (outcome measure) in an extraction table and grouping them with all eligible studies for that synthesis. Terminology was harmonized where necessary.

Results are presented in a descriptive manner in absolute (case number) and relative proportions accompanied by a corresponding table. Due to the heterogeneity of different studies, we refrained from a meta-analysis. If data were too heterogeneous to be summarized as a whole, we conducted a division into subgroups.

### Definition of key terms

Definitions for key terms describing vestibular symptoms provided by the Bárány Society are used consistently and subsequently in this review: Dizziness is the “sensation of disturbed or impaired spatial orientation without a false or distorted sense of motion”. In contrast, vertigo was defined as the “sensation of self-motion when no self-motion is occurring or the sensation of distorted self-motion during an otherwise normal head movement.” [5]. For the acute vestibular syndrome (AVS), the authors adopted the broader International Classification of Disease (ICD-11) consensus, which defines AVS as a clinical syndrome of acute-onset, continuous vertigo, dizziness, or unsteadiness lasting days to weeks, where symptoms of vestibular dysfunction (e.g., nystagmus, vomiting, motion intolerance) are generally seen, but non-mandatory by definition [8]. However, due to the importance of nystagmus in the validation of the HINTS algorithm [8], some reports deemed (spontaneous or gaze-evoked) nystagmus a mandatory symptom. Those studies are marked as AVS(+ nystagmus).

## Results

### Search results

**Fig. 1:**
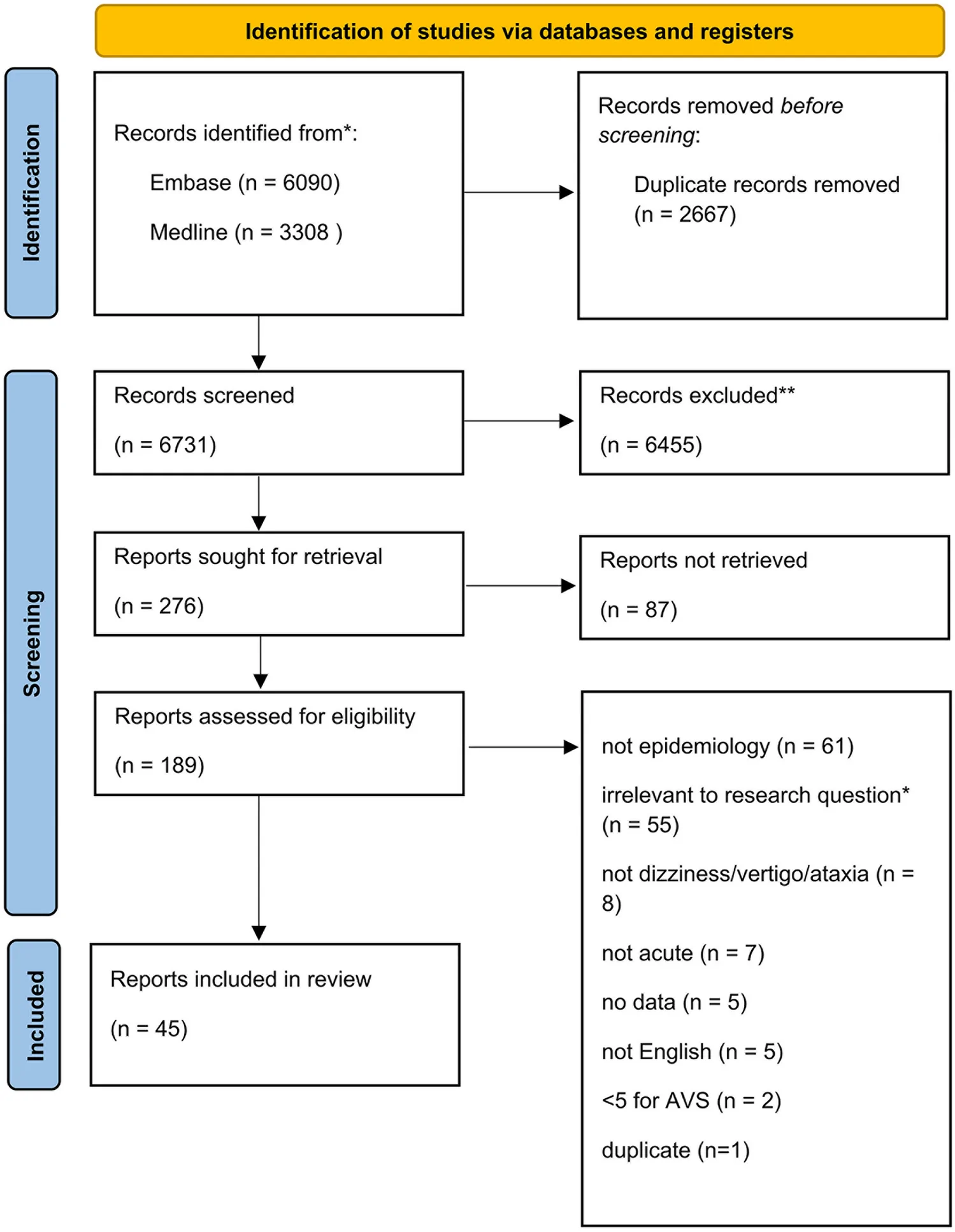
Prisma flow chart *Research questions were epidemiologic data on (1) dizziness in the emergency department, (2) acute vestibular syndrome among dizzy patients, (3) stroke among AVS presentations, or (4) specific underlying diagnoses among AVS patients prisma flow chart adapted from [60]

Our search identified 6731 unique citations, of which 6455 (95.9%) were excluded at the abstract level (see Fig. 1 – PRISMA flow chart). We sought to examine 276 full manuscripts (this included 0 articles identified by hand search) and retrieved 189 reports (out of the 87 non-retrievable reports 28 were supplemental material, 19 were available only as abstracts, 16 had no search results, 7 were meeting reports, and 17 had other reasons). After initial screening, there were a total of 68 disagreements (24.6%) about study inclusion for the two reviewers (FAC and AAT (search period: 2000–2024), kappa = 0.50 (95% confidence interval: 0.45–0.56). These differences were resolved by discussion and—if needed—adjudication by a third reviewer. Overall agreement on reason for exclusion was 96%. We demanded concordance on reason for full-text exclusion and resolved differences by discussion.

Among all 144 full-text manuscripts excluded (76.2%), the distribution of reasons for exclusion was as follows: were not about frequency/differential diagnosis or clinical presentation (42.4%), did not report data to the research question (38.2%, see Prisma flow chart), did not include data about vertigo, dizziness or imbalance (5.6%), were not acute (< 72 h) (4.9%), contained no original data (3.5%), were not in English (3.5%), had fewer than 5 subjects studied (1.4%), or were duplicates (0.7%) (see Fig. 1).

### Study characteristics

The final analysis included 45 reports in design predominantly cross-sectional (33), cohort-based (7), descriptive (4), or nonrandomized controlled trials (1). Recruitment was consecutive in 11 studies and non-consecutive in 7 studies, whereas 27 studies did not report further recruitment details. Included were articles from all continents excluding Africa and Antarctica, and two were multicentric.

In 27 studies, patients were selected based on the presence of a syndrome (i.e., acute vestibular syndrome), whereas 17 studies included patients based on specific symptoms (mainly vertigo, dizziness, or imbalance), and one study considered patients based on specific diagnoses (such as ischemic stroke or acute unilateral vestibulopathy).

Either Head Impulse, Nystagmus, and Test of Skew (HINTS) or HINTS plus (HINTS protocol with additional testing for hearing loss) [9] examination was reported in 17 studies. The ABCD2 score (clinical stroke predicting tool) was assessed in 3 studies. STANDING (SponTAneous Nystagmus, Direction, head Impulse test, and standiNG) [8] was assessed in one study.

25 out of 45 studies used some form of brain MRI as the reference standard for diagnosis, of which two did not report the use of diffusion-weighted imaging (DWI) sequences. Among studies with MRI-DWI, 15 fulfilled the “gold standard” of a delayed MRI-DWI (i.e., obtained more than 48 h after symptom onset [10]), five used an < 48 MRI-DWI, and three did not report a time frame. 16 studies used other modalities of confirming diagnosis (including MRI or CT, CT only, elective imaging, clinical suspicion, or diagnosis abstracted from medical charts), while four studies did not report a reference standard at all.

Patients were assessed in the ED in 32 studies, and either in the ED or in a dedicated vertigo clinic in one report. Other locations included the neurology department (three studies, including stroke centers) and the ENT department (1 study). Eight studies did not report the location of the assessment. The study duration ranged from one week to 168 months, with an average of a 48-month duration among all studies.

Risk of bias assessment through the adapted QUADAS-2 tool was judged as low (*n* = 11) or “high”/ “unclear” in one (*n* = 5), two (*n* = 2), three (*n* = 3), or more than three (*n* = 24) of the seven QUADAS-2 bias/applicability categories (see Appendix 3, Table S3-2).

On average, a study had 10 “low risk”, five “unclear”, and one “high risk” domain. Further characteristics of all included reports can be found in Appendix 3 (Table S3-1).

### Dizziness among ED visits

A total of 14 studies ([11–24]) reported the proportion of dizzy patients among all ED presentations. The proportion of dizziness in the ED varied from 0.8 to 5.5% across all studies (see Table 1). Combined, these studies identified 194′390 dizzy patients among 5′629′264 ED encounters, corresponding to a proportion of 3.45%. Three population-based studies ([13, 14, 17]) and two cross-sectional studies ([12, 22]) reported visits (i.e., repeat visits by the same patient are separately counted). The remaining nine ([11, 15, 16, 18–21, 23, 24]) studies reported on patients but did not indicate whether repeat visits by the same patient were excluded or included.

**Table 1:** Dizziness presentations among ED visits

Citation name	Counting visits or patients?	ALL Dizzy	ALL ED	Proportion
Mármol-Szombathy [11]	Patients	1′233	156′972	0.79%
Navi et al. [12]	Visits	907	113′375	0.80%
Kerber et al. [13]^a, b, c^	Visits	25,867	1′034′758	2.50%
Ljunggren et al. [14]^c^	Visits	2552	101′970	2.50%
Idil et al.[15]	Patients	33′615	1′190′857	2.82%
Royl et al.[16]	Patients	124	4′132	3.00%
Newman-Toker et al. [17]^b.c^	Visits	9472	287′030	3.30%
Cheung et al.[18]	Patients	413	11′319	3.65%
Lam et al.[19]	Patients	104	2′594	4.01%
Ota et al. [20]^d^	Patients	54′190	1′346′457	4.02%
Jahn et al.[21]	Patients	818	19′345	4.23%
Chang et al.[22]	Visits	41′342	926′488	4.46%
Kogej et al.[23]	Patients	2′958	54′975	5.38%
Kim et al.[24]	Patients	20′795	378′992	5.49%
All studies		194′390	5′629′264	3.45%
All studies counting patients		156′499	4′205′506	3.72%

Abbreviations: ED = Emergency department

^a^Numbers to be read in thousands

^b^Using publicly available data from National Hospital Ambulatory Medical Care Survey (NHAMCS):

Kerber et al. [13] using data from 1995–2004, Newman-Toker et al.[17] using data from 1993–2005

^c^Population-based studies

^d^Patients had to be transported by ambulance

Dizziness is a highly subjective symptom, and its documentation relies solely on patient history and cannot be disproven by clinical examination. Consequently, we accepted slight differences in documentation methods and case identification across studies. However, all studies classified a patient as dizzy when presenting to the triage nurse or physician with dizziness as the primary complaint. In addition, studies included the presenting symptoms of vertigo, unsteadiness, and/or imbalance within their dizziness classification.

### AVS among dizziness presentations

Seven studies ([25–31]) assessed AVS among ED presentations for dizziness after exclusion of three studies based on the same patient database (DETECT-Trial [32–34]) and a Swedish study [14] that overlapped in sample with Sandlund et al. [28]. In total, 1′294 AVS cases out of 12′429 dizziness presentations were reported, equating to a proportion of 10.4% with study specific proportions ranging from 1.3% to 31.6%. Table 2 displays all proportions listed and in addition the different definitions of AVS used in the studies. Notably, AVS definitions were inconsistent across included studies. Criteria were based on duration of vertigo (cutoff of > 24 h), mandatory nystagmus, threshold of symptoms, or specific symptomatic constellations.

**Table 2:** AVS among dizziness presentations

Study name	AVS	Dizzy	Proportion	AVS definition (summarized)
Dmitriew et al. [25]	39	3109	1.3%	Mandatory documentation of nystagmus and unsteady gait
Thomas et al. [26]	133	3150	4.2%	Dizziness or vertigo lasting more than 24 h associated with vestibular symptoms
Carmona et al. [44]	114	1218	9.4%	Not reported but citing 2015 Bárány consensus on AVS^b^
Sandlund et al. [28]^a^	183	1487	12.3%	Dizziness with at least two symptoms of nystagmus, nausea or vomiting, gait or balance disturbance or discomfort with head movement
Comolli et al. [29]^a^	303	1535	19.7%	Continuous dizziness with features of ongoing vestibular dysfunction
Liu et al. [30]	329	1320	24.9%	Ongoing dizziness within 72h with at least two symptoms of nystagmus, nausea, gait disturbance or discomfort with head movement. Additionally, no obvious cause for such symptoms
Machner et al. [31]	193	610	31.6%	Documentation of new spontaneous vertigo, dizziness or imbalance lasting over 24 h
All studies	1′294	12′429	10.4%	

Abbreviations: AVS = Acute vestibular syndrome

^a^ Studies counted visits instead of patients

^b^ “AVS comprises diseases and disorders that usually manifest themselves with a single episode of sudden onset of vestibular symptoms and signs: vomiting, nystagmus and postural instability” citing Bisdorff et al.[1]

### Stroke among AVS populations

A total of 25 studies [9, 14, 26, 27, 29–31, 35–52], excluding seven studies reporting on the same cohort, reported newly diagnosed stroke cases out of a pool of AVS patients, with stroke diagnoses ranging from 3 to 59% out of all patients (see Table 3). According to these studies, 979 new strokes occurred among 4,684 AVS patients, corresponding to an overall proportion of 21%. This estimate should be interpreted with caution because of substantial heterogeneity in AVS definitions and in the inclusion and exclusion criteria applied across studies.

**Table 3:** Stroke prevalence in different AVS populations

Study name	AVS definition of base population	Strokes^a^	Total AVS	Proportion of strokes (%)
Casani et al. [35]	Isolated AVS	11	392	3%
Kim et al. [36]	Isolated AVS (+ nystagmus)	23	468	5%
Ljunggren et al. [14]	AVS	41	408	10%
Kerber et al. [37]	AVS (+ nystagmus)	29	271	11%
Doijiri et al. [38]	Isolated AVS	15	118	13%
Siepmann et al. [39]	AVS (+ nystagmus)	4	30	13%
Tong et al. [40]	AVS or EVS	68	502	14%
Gerlier et al. [41]	AVS or EVS	43	312	14%
Thomas et al. [26]	AVS	20	133	15%
Gerlier et al. [42]	AVS	49	300	16%
Yebra González et al. [43]	AVS	18	97	19%
Comolli et al. [29]	AVS (+ nystagmus)	77	303	25%
Choi et al. [46]	transient AVS	23	86	27%
Nham et al. [52]	AVS or transient AVS	62	180	34%
Carmona et al. [27]	AVS (+ nystagmus)	30	81	37%
Carmona et al. [44]	AVS	42	114	37%
Machner et al. [31]	AVS (+ nystagmus)	14	38	37%
Nham et al. [45]	AVS	79	208	38%
Lemos et al. [47]	AVS (+ nystagmus)	12	30	40%
Chen et al. [48]	Isolated AVS	10	24	42%
Liu et al. [30]	AVS	52	121	43%
Sankalia et al. [49]	AVS^b^	35	75	47%
Kattah et al. [50]	AVS^b^	25	52	48%
Kattah et al. [51]	AVS	84	151	56%
Newman-Toker et al. [9]	AVS (+ nystagmus) + 1 CV risk factor	113	190	59%

Abbreviations: AVS = Acute vestibular syndrome; EVS = Episodic vestibular syndrome; (+ nystagmus) = Signifying mandatory nystagmus as inclusion criteria

^a^ Transient ischemic attack excluded if reported separately

^b^ Did not report if nystagmus was mandatory or not

To facilitate more meaningful comparisons, the studies were subdivided into methodologically more homogeneous subgroups and newly diagnosed strokes were pooled within these groups. The highest stroke proportion (59%) was reported in a diagnostic-accuracy study of HINTS ([9]) that required AVS in combination with at least one vascular risk factor (smoking, hypertension, diabetes, hyperlipidemia, atrial fibrillation, eclampsia, hypercoagulable states, recent cervical trauma, prior stroke, or myocardial infarction) (see Table 4). Eight studies ([14, 26, 27, 30, 42, 43, 45, 51]) that met ICD-11 AVS criteria (continuous vestibular symptoms but rendering nystagmus non-mandatory) were grouped together and resulted in 385 strokes among 1,532 patients resulting in a pooled proportion of 25%.

**Table 4:** Pooled stroke prevalence in different AVS populations

Inclusion criteria (n = studies)	Strokes	Base population	Stroke proportion
Isolated AVS [35, 36, 38, 48]	49	1002	5%
All studies (25 studies)	979	4684	21%
AVS (+ nystagmus) [29, 31, 36, 37, 39, 47]^a^	166	753	22%
AVS with non-mandatory nystagmus (= ICD-11 criteria) [14, 26, 30, 42–45, 51]^b^	385	1532	25%
AVS + 1stroke risk factor^c^ [9]	113	190	59%

Abbreviations: AVS = Acute vestibular syndrome

^a^ Excludes studies requiring a cardiovascular risk factor

^b^ Excludes studies requiring obligatory nystagmus, isolated AVS, transient AVS, EVS or not reporting if nystagmus was required. (= fitting ICD-11 criteria)

^c^ For stroke risk factors see text

In comparison, six studies ([29, 31, 36, 37, 39, 47] defining AVS strictly with concurrent nystagmus (referred to as “AVS + nystagmus” group) had 166 strokes in 753 patients (22%) resulting in a similar number as in the subgroup of studies where nystagmus was non-mandatory. The lowest pooled proportion of stroke with 5% was observed in four studies ([35, 36, 38, 48]) assessing isolated AVS (= iAVS). Those studies were only included if explicitly reporting excluding any focal neurological deficits, such as hemiparesis, aphasia, ataxia, or paresis.

Six studies ([27, 34, 36, 44, 45, 50]) reported specific infarction locations, and 272 stroke cases. One study reported explicitly only posterior vascular strokes [45] and one [34] reported on the data set of Comolli et al. [29]. Infarctions were distributed as follows: posterior inferior cerebellar artery (PICA) territory predominated with 154 cases (36.15%), followed by anterior inferior cerebellar artery (AICA) with 39 cases (8.39%) and basilar artery (BA) including perforators with 30 cases (6.4%). Other locations included two or more vascular territories in 22 (4.3%), the middle cerebral artery (MCA) in 11 cases (4.0%, one case associated with vestibular migraine), and the posterior cerebral artery (PCA) or the superior cerebellar artery (SCA) in 8 (1.49%) and 7 (1.29%) cases, respectively. Noteworthy, no information on the frequency of vertebral artery dissection (e.g., as an underlying cause of PICA strokes) was provided in the studies included.

### Specific diagnoses among AVS populations

Ten studies ([9, 26, 27, 29, 43, 44, 47, 49, 52, 53]) provided etiological diagnoses for 1,410 AVS patients (1418 diagnoses in total as one study permitted multiple assignments). Diagnoses were systematically categorized according to presumed etiology into three groups: peripheral vestibular, central vestibular, or non-specific (undetermined/unclassifiable). This categorization approach was consistent across the studies included though the specific diagnostic criteria and the terminology used to assign patients within these categories varied considerably.

### Peripheral vestibular causes

Peripheral vestibular etiologies accounted for 624 diagnoses (44.0% of total). Acute unilateral vestibulopathy was the predominant diagnosis, comprising 545 cases (87.3% of peripheral diagnoses, 38.4% of all diagnoses). The remaining peripheral cases were distributed among Meniere’s disease (36 cases, 5.8% of peripheral diagnoses), inflammatory conditions including labyrinthitis and otitis media (27 cases, 4.3%), benign paroxysmal positional vertigo (14 cases, 2.2%), and Ramsay Hunt syndrome (2 cases, 0.3%).

### Central vestibular causes

Central nervous system etiologies were identified in 563 cases (39.7% of total diagnoses). Cerebrovascular events—comprising ischemic stroke, hemorrhagic stroke, and transient ischemic attack—represented the largest subgroup at 432 cases (76.7% of central diagnoses, 30.5% of all diagnoses). Vestibular migraine accounted for 72 cases (12.8% of central diagnoses, 5.1% of all diagnoses). A notable proportion of patients was assigned to the central category without reporting a specific diagnosis: other unspecified central causes were recorded in 14 cases (2.5% of central diagnoses). A group of 8 cases (1.4% of central diagnoses) was of "unknown central etiology", where the corresponding studies assigned a case to the central group while reporting to not know the specific diagnoses. These patients remained within the central classification despite the absence of definitive diagnostic confirmation. Other diagnoses can be seen in Table 5.

**Table 5:** Specific diagnoses among an AVS population of 10 studies

Diagnostic Category	Diagnosis	Patients	Percentage
Peripheral	Acute unilateral vestibulopathy	545	38.43%
	Meniere’s Disease	36	2.54%
	Inflammatory processes^a^	27	1.90%
	BPPV	14	0.99%
	Viral^b^	2	0.14%
		**624**	44.01%
Central	Vascular (stroke, TIA)^c^	432	30.47%
	Vestibular migraine	78	5.50%
	Multiple Sclerosis	18	1.27%
	Other central causes^d^	14	0.99%
	Neoplastic	9	0.63%
	Unknown	8	1.42%
	Vitamin deficiency^e^	4	0.28%
		**563**	39.70%
Non-specific	Other^f^	166	11.71%
	Unknown	62	4.37%
	Drugs	3	0.21%
		**231**	16.29%
Total diagnosis		1418	100%

Bold numbers are totals for the sections (peripheral, central, non-specific)

Abbreviations: AUV = Acute Unilateral Vestibulopathy; MD = Meniere's Disease; BPPV = Benign Paroxysmal Positional Vertigo; MS = Multiple sclerosis; VM = Vestibular Migraine

Further diagnoses, N = Patients

^a^20 labyrinthitis, 7 otitis media

^b^Ramsay Hunt syndrome

^c^includes all forms of stroke; ischemic, hemorrhagic and TIA

Listing TIA separate was not possible due to mixed reporting with other stroke forms

^d^certain studies did not further differentiate into specific diagnoses

^e^4 Wernicke’s Encephalopathy

^f^includes functional, psychogenic, uncertain and other non-specific causes like hypoglycemia, high blood-pressure

### Non-specific/undetermined causes

A substantial proportion of diagnoses—231 cases (16.3% of total)—were classified as non-specific due to undetermined or unclassifiable etiology. Among these, 166 cases (72% of this subgroup, 11.7% of all diagnoses) included functional disorders, psychogenic symptoms, and/or systemic conditions, such as hypoglycemia and hypertensive episodes. In addition, 62 cases (27% of this subgroup, 4.4% of all diagnoses) remained completely undiagnosed and could not be assigned to either the central or peripheral categories nor ruled out a vestibular involvement. Finally, 4 cases (1.3% of this subgroup, 0.2% of all diagnoses) were associated with medication-related effects without specification of the causative agent.

## Discussion

In our systematic review, dizziness was the main presenting symptom in more than 3 out of 100 emergency patients and had a frequency like chest pain (3.2%) as determined in a Spanish cohort [54]. The lowest (0.8%) and the highest proportion (5.5%) show a sevenfold difference. These differences might be based on variations in the EHR (= Electronic Health Records) review. Kim et al. [24] identified patients by reviewing EHRs for a primary complaint of dizziness or vertigo recorded at triage or presentation. In contrast, Màrmbol‑Szombathy et al. [11] used ICD‑9 diagnostic codes for dizziness or vertigo to select eligible cases. Ota et al. [20] counted only ambulance-transported patients, and Newman-Toker et al. [17] used a nationwide database.

AVS presentations among dizzy patients varied by a large margin from study to study (1.3–31.6%, a 24-fold difference) with an average of 10%. A primary contributor to this difference might lie in standardized patient–clinician-aligned terminology for vertigo, dizziness, and AVS definitions. Patients experience and describe vertigo/dizziness subjectively, often conflating the sense of a spinning motion with dizziness, light-headedness, and unsteadiness. Surveys have shown that most patient descriptions are inconsistent and unreliable, and there is a weak correlation between symptom and illness [55].

Moreover, the interchangeable use of terms such as “dizziness,” “vertigo,” and "unsteadiness" on the physician side further undermines comparability across studies. Currently, there is no generally accepted definition of AVS (ICD-11 definition vs. Definition by the Bárány Society), and the use of clear consensus definitions remains limited [56]. Despite a focused search for studies explicitly addressing “acute vestibular syndrome” rather than general vertigo, substantial heterogeneity in AVS definitions persisted, reflecting the current lack of standardized diagnostic criteria in both clinical and research settings. We strongly recommend resolving discrepancies between concurring definitions of AVS and developing a consensus definition for future clinical and research use. While for peripheral vestibular causes of acute and prolonged vertigo/dizziness, a nystagmus is almost always seen. This is less often the case for central causes of acute/prolonged vertigo and dizziness, with nystagmus (either spontaneous or gaze-evoked) lacking in up to 50% of cases as shown in one recent study [56]. These patients are then referred to as suffering from an acute imbalance syndrome (AIS). Likewise, for other non-vestibular causes of acute prolonged dizziness [57], a nystagmus will be the exception.

From the viewpoint of further optimizing the diagnostic approach to the acutely dizzy patient, the use of dedicated eye movement testing as promoted by the HINTS must be restricted to suitable patients. Importantly, the HINTS have been validated in patients with an AVS + nystagmus only. If cases without nystagmus are tested as well, there is a substantial risk to classify non-vestibular causes (e.g., severe anemia or hypoglycemia) as central vestibular since HINTS will demonstrate a false-central pattern as the head-impulse test will be normal and no skew deviation or gaze-evoked nystagmus will be seen. Thus, for guiding the clinician in the diagnostic workup of the acutely dizzy patient, we recommend keeping nystagmus (spontaneous or gaze-evoked) as part of the AVS definition. Patients suffering from acute and prolonged vertigo/dizziness without any nystagmus will then be categorized as AIS.

### Etiology of AVS

Nearly one in two patients suffer a peripheral vestibular cause while central causes were linked to 39.7% of cases. Vascular events accounted for nearly one-third of all diagnoses (31%), a proportion that is considerably higher than the stroke proportion reported in the overall AVS population (see Stroke in AVS). This likely reflects a selection bias as many of the included studies were conducted in tertiary stroke centers or enriched their samples with high-risk for vascular events patients, thereby over-representing cerebrovascular causes.

Notably, a substantial proportion of patients lacked a specific etiological diagnosis and were classified only within one of the three overarching groups (peripheral, central, or non-specific) without further diagnostic detail. This pattern reflects both the inherent diagnostic limitations of the ED-setting and marked heterogeneity in diagnostic workups and classification schemes across studies. The “unknown central cause” and “unknown non-specific” categories together accounted for 70 cases (4.9% of all diagnoses), suggesting a diagnostic ceiling even in well-resourced environments and underscoring the need for improved diagnostic algorithms or biomarkers to distinguish vestibular syndromes of differing etiologies.

In addition, benign paroxysmal positional vertigo (BPPV), Menière’s disease (MD), and vestibular migraine (VM)—prototypical episodic vestibular syndromes—were recorded in 128 cases (9%). Their presence in AVS cohorts illustrates the difficulty of differentiating continuous from episodic vertigo in the acute care setting.

Conversely, prolonged or clustered attacks of MD or VM may mimic an acute continuous vestibular syndrome, further complicating bedside classification and highlighting the need for targeted education and structured diagnostic pathways for ED physicians. Due to the lack of biomarkers and diagnostic criteria for vestibular migraine requiring at least 5 episodes of vertigo or dizziness lasting between 5 min and 72 h, the true incidence of central AVS due to vestibular migraine is likely underestimated.

An unexpected finding is the substantial number of cases classified under non-specific or physiologically implausible diagnoses, including metabolic disturbances (e.g., hypoglycemia) and hypertensive episodes. While these conditions may produce general dizziness or altered sensorium, they lack a clear pathophysiological basis for producing sustained nystagmus or ocular motor abnormalities as observed in AVS (as reviewed recently [57]).

Stressful conditions in the ED may impede detailed history-taking and careful positional and ocular motor testing, leading clinicians to misclassify patients (i.e., classifying a dizzy patient without nystagmus as AVS subsequently leading to a false-positive HINTS result) as suffering from AVS. Importantly, in the recently updated, third version of the Guideline for Reasonable and Appropriate Care in the Emergency Department (GRACE-3) on adult patients with acute dizziness and vertigo in the ED, evidence-based recommendations on their management are provided [58]. Thus, this guideline is an important contribution to improving the quality of care for the acutely dizzy patient in the ED.

### Stroke in AVS

Stroke had among 25 studies a prevalence of 21%, a rate very similar to an estimation proposed already 15 years ago, where strokes were assumed to occur in 25 ± 15% of all AVS cases [59]. The presence of isolated AVS decreased the probability of a stroke fourfold in comparison with non-isolated AVS. This finding contrasts with a prior meta-analysis reporting a higher rate of central causes, particularly stroke, in patients with iAVS [3]. Upon closer comparison, this discrepancy likely stems from differences in case definitions: in our systematic review, studies were only categorized as iAVS if the term “isolated acute vestibular syndrome” was explicitly used to define the study population. Also, there might be a selection bias as iAVS strokes are harder to detect clinically and even an early MRI-DWI might be false-negative with subsequent discharge from the hospital. Future research should aim to validate these observations through profound statistical evaluation. Nevertheless, the residual 5% stroke rate in iAVS remains clinically relevant, necessitating diagnostic evaluation to exclude cerebrovascular events.

Nystagmus in a patient seems to be of less diagnostic value as studies fitting ICD-11 criteria and studies applying a stricter definition of a mandatory nystagmus are both similar in stroke proportion (25% vs 22%). However, it could be the case that both patient populations were similar as physiologically a true vertigo originating from the vestibular system should inherently cause a nystagmus. Therefore, ICD-11 strokes should have had nystagmus as well, but our studies did not report how many patients had concurrent nystagmus with their vertigo symptoms.

In addition, this might be underlining the fact that not nystagmus itself is of importance, but the type of nystagmus. Similarly, in the HINTS examination, a direction-changing, non-suppressible nystagmus indicates a stroke and not the presence of any nystagmus [2]. In clinical practice, however, this distinction adds a layer of complexity as accurate classification of nystagmus type requires both expertise and systematic assessments.

## Limitations

Our systematic review has its power in the holistic inclusion of 45 studies representing the current body of evidence, summarizing its epidemiological findings. It can be considered a lexicon or database scanning current literature. This approach limits statistical findings to a descriptive manner since most studies are heterogeneous in design (study type, inclusion and exclusion criteria), definitions (defining AVS, correlating AVS with a stroke lesion), skills of the assessing physician standard of diagnostics, and follow-up times. Moreover, due to the scarcity of targeted AVS research, we were often compelled to abstract data from studies in which the relevant information was a secondary or non-primary objective—for example, extracting epidemiological or timing data from studies designed primarily to assess diagnostic accuracy. This reliance on secondary data extraction introduces a risk of information bias as the variables of interest may not have been uniformly or systematically collected across studies. Additionally, the incomplete or selective reporting of such secondary outcomes may have contributed to underreporting, further limiting the reliability of our review’s findings. Quantitative analysis of the results was not feasible, and this study had to refrain from meta-analysis.

Thus, the range of patient populations included in our systematic review was very broad, reaching from a symptom-based presentation (i.e., acute vertigo or dizziness) to a syndromal diagnosis (i.e., acute vestibular syndrome) to specific diseases (such as AICA strokes). As a result, the level of granularity by which diagnoses were made and which diagnostic pathways were applied to these patients varied substantially. Both very broad inclusion criteria and narrow criteria (with e.g., a focus on vertebrobasilar stroke (as confirmed on MRI-DWI) vs. acute unilateral vestibulopathy in some studies) may have increased the risk for missed diagnoses. This includes especially non-structural central causes of AVS, such as vestibular migraine, intoxications, Wernicke’s encephalopathy, or paraneoplastic syndromes presenting with subacute-onset ataxia.

While the focus of our systematic review was on vestibular symptoms, some of the studies also reported on new-onset unilateral hearing loss (mostly in the context of bedside testing for subtle ocular motor signs (HINTS) in combination with hearing loss (“plus” sign)). In most cases, these were AICA strokes. However, combined audio-vestibular symptoms of acute-onset may also be due to an isolated labyrinthine stroke that cannot be detected with established MRI-DWI protocols or due to a first episode of Menière’s disease. More research is needed on combined acute audio-vestibular syndromes, including with regard to their epidemiology and clinical presentation.

## Conclusion

Dizziness, as a symptom in the ED, is well documented in diverse reports having a small to very large sample size, making up 0.8–5.5% of all ED consultations. About one in ten patients with acute dizziness suffer from AVS and about 21% of them have a stroke as an underlying cause. Nystagmus seems to be of less importance in predicting stroke prevalence, while the absence of other focal neurological deficits (i.e., iAVS) lowers stroke prevalence substantially by about fourfold to 5%. With non-specific diagnoses only or lack of any diagnosis after diagnostic workup in about 16% of all AVS cases included in our review, this emphasizes the need for improvement of the diagnostic approach in the ED and for harmonizing current AVS definitions. Potential strategies to do so include training ED physicians in the application of HINTS / STANDING and targeted history-taking, implementing quantitative and standardized assessments of (selected) acutely dizzy patients using video-oculography and offering teleconsultations by neuro-otology experts for those hospitals that lack sufficient inhouse resources. In future research studies, more emphasis should be placed on the implementation of up-to-date AVS definitions and to investigate the clinical outcomes associated with these diagnostic patterns.

## Supplementary Information

Below is the link to the electronic supplementary material.

Supplementary file1 (DOCX 29 KB)

Supplementary file2 (DOCX 38 KB)

Supplementary file3 (DOCX 269 KB)

## Data Availability

Source data used for the systematic review will be made available to others upon request to the corresponding author.
