# Supplementary material for: The epidemiology and clinical presentation of acute vestibular syndromes: a systematic review of the literature
Source: J Neurol. 2026 Mar 2;273(3):173. doi: 10.1007/s00415-026-13720-5 (PMC13676544; doi:10.1007/s00415-026-13720-5)
Supplement: Supplementary file 1 [file 415_2026_13720_MOESM1_ESM.docx]

# **Appendix 1- electronic search strategy, coding-scheme for the systematic review and data analysis**

The search strategy was designed by a neurologist with relevant domain expertise in neuro-otology (AAT). We searched MEDLINE and Embase for English-language articles, using the following strategies with the following components: (1) vertigo, dizziness or gait imbalance, (2) epidemiology, clinical presentation and differential diagnosis, (3) acute vestibular syndrome (including ischemic stroke and acute peripheral vestibulopathy) and (4) acute presentation. We also performed a manual search of reference lists from eligible articles and contacted corresponding authors where necessary. We did not seek to identify research abstracts from meeting proceedings or unpublished studies.

AGGREGATED/COMPOSITE VERSION (June 19th, 2024) [PubMed 3308 abstracts; EMBASE 6090 abstracts]

((dizz*[tiab] OR vertigo[tiab] OR vestibular[tiab] OR ataxia[tiab] OR imbalance[tiab]) AND (origin[tiab] OR spectrum[tiab] OR epidemiolog*[tiab] OR prevalence[tiab] OR frequency[tiab] OR incidence[tiab] OR distribution*[tiab] OR prodrom*[tiab] OR diagnos*[tiab] OR manifestation*[tiab] OR clinical feature*[tiab] OR symptom*[tiab] OR “physical examination”[mh] OR “medical history taking”[mh] OR physical exam*[tiab] OR “diagnostic tests, routine”[mh] OR dizziness/physiopathology[mh] OR vertigo/physiopathology[mh] OR dizziness/diagnosis[mh] OR vertigo/diagnosis[mh]) AND (thiamine[tiab] OR multiple sclerosis[tiab] OR intoxicat*[tiab] OR vestibulopathy[tiab] OR labyrin*[tiab] OR vestibular neuritis[tiab] OR vestibular neuronitis[tiab] OR vestibular syndrome[tiab] OR cerebrovascular[tiab] OR stroke*[tiab] OR cerebellar[tiab] OR hemorrhag*[tiab] OR haemorrhag*[tiab] OR vertebrobasilar insufficiency[tiab] OR TIA[tiab] OR transient ischemic attack[tiab]) AND (acute[tiab] OR new-onset[tiab] OR emerg*[tiab] OR sudden[tiab]) AND (english[la]) AND 2000:2024[dp])

DISAGGREGATED VERSION [to demonstrate overall structure]

(dizz*[tiab] OR vertigo[tiab] OR vestibular[tiab] OR ataxia[tiab] OR imbalance[tiab])

AND

(origin[tiab] OR spectrum[tiab] OR epidemiolog*[tiab] OR prevalence[tiab] OR frequency[tiab] OR incidence[tiab] OR distribution*[tiab] OR prodrom*[tiab] OR diagnos*[tiab] OR manifestation*[tiab] OR clinical feature*[tiab] OR symptom*[tiab] OR “physical examination”[mh] OR “medical history taking”[mh] OR physical exam*[tiab] OR “diagnostic tests, routine”[mh] OR dizziness/physiopathology[mh] OR vertigo/physiopathology[mh] OR dizziness/diagnosis[mh] OR vertigo/diagnosis[mh])

AND

(thiamine[tiab] OR multiple sclerosis[tiab] OR intoxicat*[tiab] OR vestibulopathy[tiab] OR labyrin*[tiab] OR vestibular neuritis[tiab] OR vestibular neuronitis[tiab] OR vestibular syndrome[tiab] OR cerebrovascular[tiab] OR stroke*[tiab] OR cerebellar[tiab] OR hemorrhag*[tiab] OR haemorrhag*[tiab] OR vertebrobasilar insufficiency[tiab] OR TIA[tiab] OR transient ischemic attack[tiab])

AND

(acute[tiab] OR new-onset[tiab] OR emerg*[tiab] OR sudden[tiab])

AND humans[mh]

AND english[la]

AND 2000:2024[dp]

**For Embase (search performed June 19^th^ 2024, 6090 citations):**

(dizz*:ab,ti OR vertigo:ab,ti OR vestibular:ab,ti OR ataxia:ab,ti) AND (origin:ab,ti OR spectrum:ab,ti OR epidemiolog*:ab,ti OR prevalence:ab,ti OR frequency:ab,ti OR incidence:ab,ti OR distribution*:ab,ti OR prodrom*:ab,ti OR diagnos*:ab,ti OR manifestation*:ab,ti OR ‘clinical features’:ab,ti  OR ‘clinical feature’:ab,ti OR symptom*:ab,ti OR ‘physical examination’/de OR ‘physical exam’:ab,ti OR  'diagnostic test'/de OR ‘diagnostic tests, routine’/de OR ‘dizziness/physiopathology’/de OR ‘vertigo/physiopathology’/de OR ‘dizziness/diagnosis’/de OR ‘vertigo/diagnosis’/de) AND  (‘thiamine‘:ab,ti OR ‘multiple sclerosis’:ab,ti OR intoxicat*:ab,ti OR ‘vestibulopathy’:ab,ti OR labyrin*:ab,ti OR ‘vestibular neuritis’:ab,ti OR ‘vestibular neuronitis’:ab,ti OR ‘vestibular syndrome’:ab,ti OR cerebrovascular:ab,ti OR stroke*:ab,ti OR cerebellar:ab,ti OR hemorrhag*:ab,ti OR haemorrhag*:ab,ti OR ‘vertebrobasilar insufficiency’:ab,ti OR tia:ab,ti OR ‘transient ischemic attack’:ab,ti OR ‘transient ischaemic attack’:ab,ti) AND (‘acute’:ab,ti OR ‘new-onset’:ab,ti OR emerg*:ab,ti OR ‘sudden’:ab,ti) AND [1-1-2000]/sd AND [English]/lim

## **Inclusion and exclusion rules for abstracts & full-text manuscripts**

**All gathered literature was subject to title/abstract screening by two independent reviewers (AAT/FAC). Abstract review coding rules are provided below. Full-text screening was applied to all citations considered eligible or possibly eligible by at least one reviewer. Two independent reviewers (AAT/FAC) determined whether full-text manuscripts are eligible and, if not, provided a reason for exclusion (see full-text review coding rules below). Differences were resolved by discussion and consensus. FAC and AAT completed a hand search of the reference lists of selected articles for additional citations. For citations identified by hand search, the full process was repeated iteratively until no additional manuscripts were found for inclusion. Inter-rater agreement on full-text inclusion was calculated using Cohen’s kappa.^1^**

## **Abstract review coding rules**

1) Coding status options are “Yes”, “No”, “Maybe”. We will review full text of “Yes” and “Maybe”.

2) Err on the side of “Maybe” if there is doubt about a “No”; this is more conservative.

3) If there is only a title, exclude it only if you feel confident; otherwise code it as “Maybe”.

4) Each "No" should be coded with a reason for exclusion.

5) Reasons for exclusion are listed below 0-7. Go through them in order from 0 to 7 for each abstract, coding the first reason for exclusion only, not multiple reasons for exclusion. Only code "0" for “not English” if you are sure it is “not English”.

6) Two independent raters (AAT/FAC) will code reason for exclusion, but we will *not* mandate agreement on exclusion reason at the abstract level.

7) Occasionally an abstract seems inappropriate for another reason. In such cases, code as “other”. There should be few abstracts coded as “other.”

## **Abstract reasons for exclusion**

| 0 | not English | manuscript is not in English |
| --- | --- | --- |
| 1 | no data | review paper; no original patient data |
| 2 | not dizziness | no reasonable prospect that the study includes data about dizziness, vertigo, imbalance/ataxia |
| 3 | not acute | no reasonable prospect that the study includes data about *acute* (<72 hours) dizziness, vertigo or imbalance |
| 4 | not epidemiology | no reasonable prospect that the study includes data about the frequency, spectrum / differential diagnosis or clinical presentation in acute central (specifically stroke) or peripheral (specifically vestibular neuritis) disorders |
| 5 | <5 cases | fewer than 5 subjects (total participants reported, including cases and controls) |
| 6 | abstract only | only abstract available (from poster presentation or talk at conference) |
| 7 | other | any other reason abstract is not included |

## **Full-text review coding rules**

1) Coding status options are “Yes” or “No”.

2) Each "No" should be coded with a reason for exclusion.

3) Reasons for exclusion are listed below 0-6. Go through them in order from 0 to 6 for each full manuscript, coding the first reason for exclusion only, not multiple reasons for exclusion.

4) Two independent raters (AAT/FAC) will code reason for exclusion, and we will mandate agreement on exclusion reason at the manuscript level.

5) Coding differences will be adjudicated or consensus will be developed through dialogue.

## **Full-text reasons for exclusion**

| 0 | not English | manuscript is not in English |
| --- | --- | --- |
| 1 | no data | review paper; no original patient data |
| 2 | not dizziness | no reasonable prospect that the study includes data about dizziness, vertigo, imbalance/ataxia |
| 3 | not acute | no reasonable prospect that the study includes data about *acute* (<72 hours) dizziness, vertigo or imbalance |
| 4 | not epidemiology | no reasonable prospect that the study includes data about the frequency, spectrum / differential diagnosis or clinical presentation in acute central (specifically stroke) or peripheral (specifically vestibular neuritis) disorders |
| 5 | <5 cases | fewer than 5 subjects (total participants reported, including cases and controls) |
| 6 | abstract only | only abstract available (from poster presentation or talk at conference) |
