# Supplementary material for: The epidemiology and clinical presentation of acute vestibular syndromes: a systematic review of the literature
Source: J Neurol. 2026 Mar 2;273(3):173. doi: 10.1007/s00415-026-13720-5 (PMC13676544; doi:10.1007/s00415-026-13720-5)
Supplement: Supplementary file 2 [file 415_2026_13720_MOESM2_ESM.docx]

## **Appendix 2 - QUADAS-2 assessment of included studies**

For included studies, two independent raters (FAC/AAT) assessed the risk of bias or applicability concerns using QUADAS-2^2^ tailored study criteria, resolving disagreements by discussion.

The QUADAS-2 tool for quality rating of diagnostic accuracy studies consists of four core domains (patient selection, index test, reference standard, and flow and timing).^2^ Risk of bias is assessed for all four domains, and applicability is assessed for the first three domains. Thus, seven items per study are assessed to rate quality of evidence. For each item, pre-specified conditions must be met to qualify for “low risk” of bias. The tool’s authors recommend review-specific tailoring of the rating criteria and process,^2^ which we did for all seven criteria (Box S1-1).

Consensus ratings for those studies that were included (n=100) are provided in Table S1-1.

### *Box S1-1. Tailored QUADAS-2 rating criteria for key domains of bias or applicability concerns*

- Patient selection (bias): Studies were rated as *low* risk if the population was well-described and unbiased. Studies were rated as *unclear* if patients were drawn from a prospective patient registry without further explanation of original selection. Studies were rated as *high* risk if patients were chosen in non-consecutive/non-random fashion or if they studied a known clinical or demographic subgroup in whom test properties of bedside findings were likely overstated (e.g., sensitivity of neurologic findings for stroke if only patients with such findings were investigated) or understated (e.g., eye movement exams applied to incorrect subpopulations of dizziness/vertigo, such as head impulse testing applied to patients with episodic, positional vertigo).
- Patient selection (applicability concerns): Studies were rated as *low* risk if they used clear, accepted definitions for vertigo/dizziness and/or AVS (Box S1-2).^3,4^ Studies were rated as *unclear* if patients were classified but without clear definitions (e.g., said to have AVS, but without defining AVS directly or referencing previously published criteria). Studies were rated as *high* risk if patients were classified with clear definitions that did not align with accepted definitions.
- Index test (bias): Studies were rated as *low* risk if bedside (index) tests were performed by subspecialists (neuro-ophthalmology, neuro-otology, or other clinicians routinely using vestibular tests [e.g., vestibular physical therapists, vestibular lab technicians]) or trained specialists (e.g., neurologists) or generalists (e.g., ED physicians) where rater skills were shown to be accurate and reliable. Studies were rated *unclear* if tests were performed by untrained specialists (general neurologists or otolaryngologists); specialty trainees (e.g., neurology or otolaryngology residents); if the training of those who performed the bedside examinations was either mixed (e.g., specialists and ED physicians); or if training or masking was not specifically reported. Studies were rated as having *high* risk if more than 20% of tests were performed by generalists who were untrained or whose test performance was of low quality or not assessed.
- Index test (applicability concerns): Studies were rated as *low* risk if bedside test techniques were described in sufficient detail to match accepted standards (e.g., “The HIT was performed by displacing the head laterally 10-20 degrees and then rapidly rotating to the center position.”) or reference was made to using techniques as defined in prior publications with sufficient details (e.g., “The HIT was performed as in Newman-Toker et al., 2008”). Studies were rated as having *high* risk if bedside test techniques were described or demonstrated (e.g., by video) to not match accepted standards or were inconsistently applied. Studies were rated as *unclear* if bedside techniques were not described sufficiently (e.g., “We performed the head impulse test”).
- Reference standard (bias): The risk of bias in the reference standard was determined using rules specific to each diagnosis (see Box S1-3).
- Reference standard (applicability concerns): Studies were rated as *low* risk if standard diagnostic criteria were used to define stroke and/or peripheral vestibular disorders, and these criteria were compatible with existing consensus definitions and guidelines.^5^ ^6-10^ Studies were rated as *unclear* if definitions were not reported. Studies were rated as *high* risk if non-standard definitions were applied.
- Flow and timing (bias): Studies were rated as *low* risk if all patients underwent the same (or an equivalently valid) reference standard in a timely manner. Studies were rated as *unclear* if details were not reported. Studies were rated as *high* risk if the reference standard varied or timing was not appropriate (e.g., if an MRI reference standard was too early or too late, lowering its sensitivity) in more than 20%.

Abbreviations: AVS – acute vestibular syndrome; ED – emergency department; HIT – head impulse test; LOE – Level of Evidence

### *Box S1-2. Consensus (ICVD) Definitions for Vestibular Syndromes (i.e., dizziness/vertigo subpopulations)*

- Episodic Vestibular Syndrome (EVS): A clinical syndrome of transient vertigo, dizziness, or unsteadiness lasting seconds to hours, occasionally days, and generally including features suggestive of temporary, short-lived vestibular system dysfunction (e.g., nausea, nystagmus, sudden falls).
- Acute Vestibular Syndrome (AVS): A clinical syndrome of acute-onset, continuous vertigo, dizziness, or unsteadiness lasting days to weeks, and generally including features suggestive of new, ongoing vestibular system dysfunction (e.g., vomiting, nystagmus, postural instability).
- Chronic Vestibular Syndrome (CVS): A clinical syndrome of chronic vertigo, dizziness, or unsteadiness lasting months to years and generally including features suggestive of persistent vestibular system dysfunction (e.g., oscillopsia, nystagmus, gait unsteadiness).

Abbreviations: ICVD – International Classification of Vestibular Disorders

### *Box S1-3. Valid reference standards for central and peripheral vestibular disorders with risk of bias*

Stroke: All strokes must have been confirmed to be acute by neuroimaging, generally by MRI-DWI, to be rated as low risk. Exceptions to the need for MRI-DWI for low risk include (1) new non-DWI MRI lesions confirmed by repeat imaging; or (2) brain hemorrhages or large ischemic strokes confirmed by CT. Furthermore, strokes must have clinically been deemed causal, rather than incidental; specifically, strokes must have been found in a brain region appropriate to the presenting clinical syndrome (generally brainstem or cerebellum). Studies that fell short of these requirements were rated as high risk, whereas studies not providing sufficient detail on neuroimaging were considered of unclear risk.

Vestibular neuritis (acute unilateral vestibulopathy): Patients must have had an AVS clinical presentation, a spontaneous or gaze-evoked nystagmus which obeyed Alexander’s law, evidence of a unilateral vestibular deficit on quantitative physiologic testing (caloric or quantified head impulse testing), and negative MRI-DWI neuroimaging in an optimal time window in order to be rated as low risk. The MRI-DWI time window from onset of AVS symptoms determined the QUADAS-2 risk of bias on reference standard: low risk (48 hours to 7 days, inclusive), unclear risk (12-48 hours or 8-14 days), high risk (otherwise). If the time windows were non-uniform across patients, no more than 20% of patients could be in a higher risk category to still maintain the lower risk category for QUADAS-2 status. If hearing loss was present (i.e., labyrinthitis), then the risk of bias was automatically unclear, unless there was direct otoscopic evidence of a suppurative middle ear disorder or clear response to antibiotic treatment. Studies that fell short of these requirements were rated as high risk, whereas studies not providing sufficient detail were considered unclear risk.

Drug intoxication: Patients must have had an AVS clinical presentation and demonstrated supra-therapeutic drug levels of centrally acting drugs known to cause neurological symptoms (i.e., anti-epileptics, neuroleptics) to be rated as low risk. Studies that fell short of these requirements were rated as high risk, whereas studies not providing sufficient detail (i.e., no drug levels or specific medication) were considered unclear risk.

Vestibular migraine: Patients must have had an AVS clinical presentation and a history of migraine headaches (according to HIS ICHD 3), meeting the clinical definition as published by the Barany Society for vestibular migraine. Studies that fell short of these requirements were rated as high risk, whereas studies not providing sufficient detail were considered unclear risk.

Internal-medicine related specific diagnoses: Patients must have had an AVS clinical presentation and demonstrated objective (i.e., lab-based or otherwise quantitative) changes in the diagnostic work-up (e.g. hypernatriemia, hypoglycemia in the blood workup or cardiac arrhythmia on ECG) to be rated as low risk. Studies that fell short of these requirements were rated as high risk, whereas studies not providing sufficient detail (i.e., no specific lab workup results or results from quantitative testing) were considered unclear risk.

Symptom-based diagnoses solely: Patients diagnosed with symptom-based diagnoses only, e.g. acute vertigo, dizziness or ataxia will always be rated as high risk since such symptom-based diagnoses will not be based on consensus diagnostic criteria.

Abbreviations: AVS – acute vestibular syndrome; DWI – diffusion weighted imaging; EVS – episodic vestibular syndrome; ICVD – International Classification of Vestibular Disorders

**References**

1.	Cohen J. A coefficient for agreement for nominal scales. *Educ Psychol Meas* 1960; **20**: 37-46.

2.	Whiting PF, Rutjes AW, Westwood ME, et al. QUADAS-2: a revised tool for the quality assessment of diagnostic accuracy studies. *Ann Intern Med* 2011; **155**(8): 529-36.

3.	Newman-Toker D. Vestibular syndrome definitions for the International Classification of Vestibular Disorders. Bárány Classification Committee Meeting on March 10-11, 2017; 2017; Berlin, Germany; 2017.

4.	Newman-Toker DE, Edlow JA. TiTrATE: A Novel, Evidence-Based Approach to Diagnosing Acute Dizziness and Vertigo. *Neurol Clin* 2015; **33**(3): 577-99, viii.

5.	Easton JD, Saver JL, Albers GW, et al. Definition and evaluation of transient ischemic attack: a scientific statement for healthcare professionals from the American Heart Association/American Stroke Association Stroke Council; Council on Cardiovascular Surgery and Anesthesia; Council on Cardiovascular Radiology and Intervention; Council on Cardiovascular Nursing; and the Interdisciplinary Council on Peripheral Vascular Disease. The American Academy of Neurology affirms the value of this statement as an educational tool for neurologists. *Stroke* 2009; **40**(6): 2276-93.

6.	Bhattacharyya N, Gubbels SP, Schwartz SR, et al. Clinical Practice Guideline: Benign Paroxysmal Positional Vertigo (Update). *Otolaryngol Head Neck Surg* 2017; **156**(3_suppl): S1-S47.

7.	Fife TD, Iverson DJ, Lempert T, et al. Practice parameter: therapies for benign paroxysmal positional vertigo (an evidence-based review): report of the Quality Standards Subcommittee of the American Academy of Neurology. *Neurology* 2008; **70**(22): 2067-74.

8.	von Brevern M, Bertholon P, Brandt T, et al. Benign paroxysmal positional vertigo: Diagnostic criteria. *J Vestib Res* 2015; **25**(3-4): 105-17.

9.	Lopez-Escamez JA, Carey J, Chung WH, et al. Diagnostic criteria for Meniere's disease. *J Vestib Res* 2015; **25**(1): 1-7.

10.	Lempert T, Olesen J, Furman J, et al. Vestibular migraine: diagnostic criteria. *J Vestib Res* 2012; **22**(4): 167-72.
