# Supplementary material for: The epidemiology and clinical presentation of acute vestibular syndromes: a systematic review of the literature
Source: J Neurol. 2026 Mar 2;273(3):173. doi: 10.1007/s00415-026-13720-5 (PMC13676544; doi:10.1007/s00415-026-13720-5)
Supplement: Supplementary file 3 [file 415_2026_13720_MOESM3_ESM.docx]

# 1) **Supplemental Source Data & Raw Results**

## **Table S3-1: Studies included in the systematic review**

| **Author** | **Data collection (study type, enrolment)** | **Study origin** | **Study time frame (months)** | **Patient inclusion criteria - general classification** | **Patient inclusion criteria? – further details** | **Clinical test batteries used** | **Reference standard to confirm diagnosis** | **Reference standard to confirm diagnosis – further details** |
| --- | --- | --- | --- | --- | --- | --- | --- | --- |
| Carmona S, et al.[1] | cross-sectional retrospective | Santa Fe, AR | 84 | syndrome | AVS | general neurologic exam Dix-hallpike Truncal ataxia HINTSplus | early MRI-DWI (<48h) | never <12h |
| Casani AP, et al.[2] | descriptive retrospective | Pisa, IT | 60 | syndrome | clinical peripheral AVS (i.e. iAVS) with stroke as etiology | general neurologic exam caloric testing | MRI (no DWI, no time frame) |  |
| Chang YS, et al.[3] | cohort retrospective | TN | 120 | symptom | major complaint dizziness, vertigo or unsteadiness (assigned by triage nurse) | not reported | not reported |  |
| Chen L, et al.[4] | cross-sectional prospective | Melbourne, AU | 12 | syndrome | iAVS | general neurologic exam HINTS | MRI-DWI (no time frame) | not reported, but abstracted from Table 2: 1-9d after symptom onset |
| Cheung CSK, et al.[5] | cohort prospective | HK | 1 | symptom | major complaint dizziness | not reported | CT-scan | "A central neurological cause for dizziness was deﬁned as present if there was a positive CT scan or a diagnosis on the CMS of transient ischaemic attack (TIA)" |
| Choi JH, et al.[6] | cross-sectional prospective consecutive | Pusan, KR | 12 | syndrome | Transient AVS defined as 1. vertigo/dizziness 2. resolution within 24h 3. no previous vertigo history | ABCD2 score HINTSplus | early MRI-DWI (<48h) | “Brain images were obtained within 24 hours from the symptom onset in 91%. The remaining 8 patients had the imaging between 1 and 4 days from the symptom onset.” |
| Comolli L, et al.[7] | cross-sectional prospective and consecutive | Berne, CH | 73 | symptom | major complaint dizziness, vertigo or unsteadiness | not reported | Other | follow-up diagnosis |
| Dmitriew C, et al.[8] | cohort retrospective | Sudbury, CA | 42 | symptom | major complaint dizziness, vertigo or unsteadiness | HINTS | Other | "Diagnoses were confirmed by computed tomography (CT), magnetic resonance imaging (MRI), neurology consult, or diagnostic codes within ICES." |
| Doijiri R, et al.[9] | cross-sectional retrospective consecutive | Osaka, JP | 120 | syndrome | isolated dizziness or iAVS | not reported | CT or MRI | "From the preferential viewpoint of our study, intensive neuroimaging examinations using CT or MRI were undertaken in all patients" |
| Gerlier C, et al.[10] | cohort prospective | Paris, FR | 12 | syndrome | AVS or EVS  (theoretically iAVS since focal neurological signs excluded) | STANDING Algorithm graded instability rating Dix-hallpike | early MRI-DWI (<48h) | “The reference test used for differentiating central from peripheral diagnoses was brain MRI-­DWI, except in two cases:  (a) atypical diagnosis of BPPV found by the senior EP and validated by the examination of an otologist through positional tests plus videonystagmography or video-­HIT, according to the standard of care in our hospital,  and (b) a MRI contraindication—­in this case, a brain CT with angiography of the circle of Willis (CTA) was used as reference standard.” |
| Gerlier C, et al.[11] | cohort prospective | Paris, FR | 16 | syndrome | iAVS | ABCD2 score HINTS STANDING Algorithm Dix-hallpike pagnini test | MRI-DWI (time frame in text) | >48h after symptom onset |
| Idil H, et al.[12] | cross-sectional retrospective | Izmir, TR | 84 | symptom | isolated dizziness, vertigo or unsteadiness | general neurologic exam HINTS HINTSplus | CT or MRI | within 48h:,  CAVE not all received reference standard (selection based on clinical criteria: ABCD2, HINTSplus or focal neurologic deficits)  "As a result of all clinical evaluations, cranial CT and/or DW- MRI studies were carried out for patients with suspicion of central dizziness."  "Neuroimaging studies carried out within 48 hours for patients presenting with isolated dizziness to the ED were evaluated." |
| Jahn K, et al.[13] | cross-sectional retrospective | Munich, DE | 12 | symptom | major complaint dizziness, vertigo or unsteadiness | not reported | Other | "As diagnoses were made in a clinical environment, they are not based on formal diagnostic criteria" |
| Kattah JC, et al.[14] | cross-sectional prospective consecutive | Peoria, USA | NR | syndrome base | AVS | general neurologic exam HINTSplus Truncal ataxia | Other | central: either positive HINTSplus and focal abnormalities or MRI-DWI  peripheral: "there was a unidirectional contra lesional horizontal nystagmus, a unilateral abnormal HIT, no skew deviation, normal MR imaging, and characteristic clinical course." |
| Kattah JC, et al.[15] | cross-sectional prospective | AR, ES, PT | NR | syndrome | iAVS | general neurologic exam HINTS Truncal ataxia | MRI (no DWI, no time frame) | "As gold standard, a 1.5 T brain MRI was used for the definitive diagnosis of vascular lesion, unless the vascular lesion could be seen in the CT." |
| Kerber KA, et al.[16] | cross-sectional prospective | Ann Arbor, USA | 40 | syndrome | AVS (patients described differently but inclusion criteria fit AVS definition) | general neurologic exam ABCD2 score HINTSplus Dix-hallpike | MRI (no DWI) >24h | "All enrolled participants were offered a research MRI of the brain if a clinical MRI was not performed or was performed within 24 hours of symptom onset and was negative for acute infarction or ICH. Research MRIs were to be performed >24 hours from symptom onset." |
| Kerber KA, et al.[17] | cross-sectional retrospective | National Survey in USA | 120 | symptom | dizziness or vertigo | not reported | not reported |  |
| Kim AS, et al.[18] | cohort retrospective | California, USA | 12 | symptom | major complaint dizziness, vertigo or unsteadiness | not reported | clinical diagnosis | patient chart diagnosis (imaging not reported) |
| Kim MB, et al.[19] | cross-sectional retrospective | Seoul, KR | 108 | syndrome | isolated AVS (iAVS) (+spontaneous nystagmus) AND stroke | general neurologic exam | MRI-DWI (no time frame) | "Upon a suspicious VNG finding of the central lesion or a neurologic sign, we immediately performed MRI (including diffusion-weighted im- ages) and magnetic resonance angiography of the vertebral and basilar arteries." |
| Kogej M, et al.[20] | cross-sectional retrospective | Bonn, DE | 22 | symptom | major complaint dizziness or vertigo | not reported | other | ICD10 code on patient electronic health record:  "To better characterize the dizziness patients and analyse the poten­tial for improvement of the MTS, they were divided into subgroups ac­ cording to the etiology of their dizziness. This subgrouping was carried out using the ICD 10 diagnosis provided during their treatment period. Four groups were classified based on literature [13,14], as follows:" |
| Korda A, et al.[21] | cross-sectional prospective not consecutive | Berne, CH | 58 | syndrome | AVS (+nystagmus) | not reported | late MRI-DWI (>72h) | "All patients received an acute MR brain scan either within 48 h in the ED or a second, delayed MRI (3–10 days after symptoms onset), if there was no acute MRI indicated based on clinical grounds or if the ﬁrst acute MRI was non-diagnostic." |
| Korda A, et al.[22] | cross-sectional prospective | Berne, CH | 68 | syndrome | AVS (+nystagmus) | general neurologic exam HINTS  caloric testing | late MRI-DWI (>72h) | "All patients received an acute MRI either within 48 h in the ED or a second, delayed MRI, if based on clinical grounds there was no acute MRI indicated or if the first acute MRI was nondiagnostic." |
| Lam JMY, et al.[23] | cross-sectional prospective consecutive | Hong Kong, HK | 0,25 | symptom | major complaint dizziness, vertigo or unsteadiness | not reported | not reported |  |
| Lemos J, et al.[24] | cross-sectional prospective consecutive | Coimbra, PT | 6 | syndrome | AVS(+nystagmus) | general neurologic exam HINTS | MRI-DWI (time frame in text) | >48h after symptom onset (except one with CT who had stroke and a pacemaker) |
| Ljunggren M, et al.[25] | cross-sectional retrospective | Umea, SE | 36 | symptom | major complaint dizziness, vertigo or unsteadiness (with subdivision into AVS) | not reported | Other | “Dizziness visits were diagnosed according to ICD-10 as stated in the final discharge re- cords with 3 exceptions:" |
| Machner B, et al.[26] | cross-sectional retrospective | Lübeck, DE | 24 | syndrome | isolated iAVS(+nystagmus) | not reported | MRI-DWI (no time frame) |  |
| Mantokoudis G, et [27]al. | cross-sectional prospective non-consecutive | Berne, CH | 58 | syndrome | AVS(+nystagmus) | not reported | late MRI-DWI (>72h) | "All patients received a second MRI 3–­10 days later unless their first MRI was diagnostic." |
| Mantokoudis G, et al.[28] | cross-sectional prospective non-consecutive | Berne, CH | 58 | syndrome | AVS (+nystagmus) | not reported | late MRI-DWI (>72h) |  |
| Màrmol-Szombathy I, et al.[29] | descriptive retrospective | Sevilla, ES | 12 | symptom | major complaint dizziness or vertigo | not reported | not reported |  |
| Michailidou E, et al.[30] | cross-sectional prospective non-consecutive | Berne, CH | 58 | syndrome | AVS(+nystagmus) | general neurologic exam | late MRI-DWI (>72h) |  |
| Morrison M, et al.[31] | cross-sectional prospective non-consecutive | Berne, CH | 58 | syndrome | AVS(+nystagmus) | general neurologic exam | late MRI-DWI (>72h) |  |
| Navi BB, et al.[32] | cross-sectional retrospective consecutive | San Francisco, USA | 36 | symptom | major complaint dizziness, vertigo or unsteadiness | not reported | other | patient chart diagnosis |
| Newman-Toker DE, et al.[33] | cross-sectional retrospective | USA | 156 | symptom | dizziness, vertigo or unsteadiness (using triage symptoms and ICD9 codes) | not reported | Other | NHAMCS data; no unique reference standard |
| Newman-Toker DE, et al.[34] | cross-sectional prospective | Peoria, USA | 168 | syndrome | AVS(+nystagmus) + 1 stroke risk factor | general neurologic exam ABCD2 score HINTS HINTSplus | late MRI-DWI (>72h) | Delayed MRI obtained, if patient had new clinical signs of infarction. |
| Nham B, et al.[35] | cohort prospective non-consecutive | Sydney, AU | 53 | syndrome | vertigo (subdivided in AVS, tAVS, EVS, CVS) | general neurologic exam Truncal ataxia | MRI-DWI (time frame in text) | >48h after symptom onset MRI for everyone excluding patients fitting AUV diagnostic criteria |
| Nham B, et al.[36] | cross- sectional prospective consecutive | Sydney, AU | 43 | symptom | vertigo or unsteadiness | general neurologic exam | MRI-DWI (time frame in text) | >48h, if not meeting diagnostic criteria of AUV "Delayed MRI brain with DWI was done at least 48 h after symptom onset, unless contraindicated in acute vestibular syndrome patients not meeting diagnostic criteria for VN outlined above." |
| Ota K, et al.[37] | descriptive retrospective | Osaka, JP | 36 | diagnosis | ICD-10 codes related to dizziness + transport by AMBULANCE (data like US Insurance data) | not reported | other | ICD10 diagnosis in patient chart |
| Royl G, et al.[38] | cross-sectional retrospective consecutive | Berlin, DE | 12 | symptom | major complaint dizziness + ER neurology consultation | general neurologic exam | clinical diagnosis | ED physician and ED neurologist diagnosis |
| Sandlund MG, et al.[39] | nonrandomized CT  prospective | Umea, SE | 48 | symptom | major complaint dizziness, vertigo or unsteadiness | general neurologic exam | other | patient chart diagnosis, coded after ICD10 |
| Sankalia D, et al.[40] | cross-sectional not reported | Pune, IN | 21 | syndrome | AVS | HINTS | late MRI-DWI (>72h) |  |
| Siepmann T, et al.[41] | cross-sectional prospective consecutive | Dresden, DE | 12 | syndrome | AVS | general neurologic exam HINTSplus | late MRI-DWI (>72h) | Early MRI with repetition if suspicion remained: "When clinical but not imaging assessment led to suspicion of stroke, MRI was repeated after 48–72 h from study inclusion." |
| Thomas JO, et al.[42] | cross-sectional prospective consecutive | Sydney, AU | 44 | syndrome | isolated AVS (iAVS) as neurological deficits needed to be excluded | general neurologic exam HINTS Dix-hallpike | MRI-DWI (time frame in text) | >48h |
| Tong D, et al.[43] | cross-sectional retrospective consecutive | Suqiang, CN | 24 | syndrome | AVS or EVS + diagnosis of ischemic stroke | not reported | MRI-DWI (time frame in text) | MRI within 24 to 72h after symptom onset |
| von Werdt M, et al.[44] | cross-sectional prospective non-consecutive | Berne, CH | 70 | syndrome | AVS(+nystagmus) | HINTS caloric testing pure tone audiometry | MRI-DWI (time frame in text) | >48h in all patients |
| Yebra González L, et al.[45] | descriptive retrospective | Madrid, ES | 31 | syndrome | AVS | general neurologic exam HINTS | clinical diagnosis | diagnosis by neurologist or ENT physician, CT or MRI only on suspicion of stroke |

Abbreviations: ABCD2 = age, blood pressure, clinical features, duration, and diabetes score; AUV = acute unilateral vestibulopathy; AVS = acute vestibular syndrome; BPPV = benign paroxysmal positional vertigo; CMS = clinical management system; CT = computed tomography; CTA = computed tomography angiography; CVS = chronic vestibular syndrome; DWI = diffusion-weighted imaging; ED = emergency department; ER = emergency room; EVS = episodic vestibular syndrome; HIT = head-impulse test; HINTS = head-impulse test, nystagmus, test of skew; HINTSplus = HINTS plus hearing test or additional central signs; iAVS = isolated acute vestibular syndrome; ICH = intracerebral hemorrhage; ICD-9/ICD-10 = International Classification of Diseases, 9th/10th revision; ICES = Institute for Clinical Evaluative Sciences; MRI = magnetic resonance imaging; MRI-DWI = brain MRI including diffusion-weighted imaging; MTS = Manchester Triage System; NHAMCS = National Hospital Ambulatory Medical Care Survey; NR = not reported; tAVS = transient acute vestibular syndrome; TIA = transient ischemic attack; VN = vestibular neuritis; VNG = video nystagmography.

## **Table S3-2: Studies risk of bias according to QUADAS-2**

| Author | Was a consecutive or random sample of patients enrolled? | Was a case-control design avoided? | Did the study avoid inappropriate exclusions? | Patient inclusion criteria | Review the index test and how it was conducted and interpreted. | Were the index test results interpreted without knowledge of the results of the reference standard? | Index test | Was there an appropriate interval between index test(s) and reference standard | Did ALL patients receive a reference standard? | Did patients receive the SAME reference standard? | Were ALL patients included in the analysis? | Flow timing Consensus | Were the reference standard results interpreted without knowledge of the results of the index test? | Is the reference standard likely to correctly classify the target condition? | Review the reference standard and how it was conducted and interpreted. | Reference Standard Consensus | Applicability: Are there concerns that the included patients do not match the review question? | Applicability: Are there concerns that the index test, its conduct, or its interpretation differ from the review question? | Applicability: Are there concerns that the TARGET CONDITION as defined by the reference standard does not match the review question? |
| --- | --- | --- | --- | --- | --- | --- | --- | --- | --- | --- | --- | --- | --- | --- | --- | --- | --- | --- | --- |
| Carmona S, et al.[1] | No; high risk | Yes; low risk | Unclear | high risk | unclear risk | Yes; low risk | unclear | Yes; low risk | Yes; low risk | Yes; low risk | Yes; low risk | low risk | Yes; low risk | Unclear | low risk | unclear | No; low risk | No; low risk | No; low risk |
| Casani AP, et al.[2] | No; high risk | Yes; low risk | Yes; low risk | high risk | unclear risk | Unclear | unclear | Yes; low risk | Yes; low risk | Yes; low risk | Yes; low risk | low risk | Unclear | Yes; low risk | unclear risk | unclear | No; low risk | No; low risk | No; low risk |
| Chang YS, et al.[3] | Unclear | Yes; low risk | Yes; low risk | Unclear | unclear risk | Unclear | unclear | Unclear | Unclear | Unclear | Yes; low risk | Unclear | Unclear | Unclear | unclear risk | unclear | No; low risk | Unclear | Unclear |
| Chen L, et al.[4] | Yes; low risk | Yes; low risk | Yes; low risk | low risk | unclear risk | Yes; low risk | unclear | Yes; low risk | Yes; low risk | Yes; low risk | Yes; low risk | low risk | Yes; low risk | Yes; low risk | low risk | low risk | Yes; high risk | Yes; high risk | Unclear |
| Cheung CSK, et al.[5] | Yes; low risk | Yes; low risk | Yes; low risk | low risk | high risk | Unclear | high risk | Unclear | Unclear | Yes; low risk | Yes; low risk | Unclear | Unclear | Unclear | unclear risk | unclear | Unclear | Unclear | Unclear |
| Choi JH, et al.[6] | Yes; low risk | Yes; low risk | Yes; low risk | low risk | low risk | Yes; low risk | low risk | Yes; low risk | Yes; low risk | Yes; low risk | Yes; low risk | low risk | Yes; low risk | Yes; low risk | unclear risk | unclear | Unclear | Unclear | No; low risk |
| Comolli L, et al.[7] | Yes; low risk | Yes; low risk | Yes; low risk | low risk | high risk | Yes; low risk | high risk | Unclear | Unclear | Unclear | Unclear | Unclear | Unclear | Unclear | unclear risk | unclear | No; low risk | Unclear | Unclear |
| Dmitriew C, et al.[8] | Yes; low risk | Yes; low risk | Yes; low risk | low risk | high risk | Unclear | high risk | Unclear | Yes; low risk | Unclear | Yes; low risk | Unclear | Unclear | Unclear | unclear risk | unclear | No; low risk | No; low risk | Unclear |
| Doijiri R, et al.[9] | Yes; low risk | Yes; low risk | Yes; low risk | low risk | unclear risk | Unclear | unclear | Yes; low risk | Yes; low risk | No; high risk | Yes; low risk | high risk | Unclear | No; high risk | unclear risk | high risk | Unclear | Unclear | Unclear |
| Gerlier C, et al.[10] | Yes; low risk | Yes; low risk | Yes; low risk | low risk | low risk | Yes; low risk | low risk | Yes; low risk | Yes; low risk | No; high risk | Yes; low risk | high risk | Yes; low risk | Yes; low risk | low risk | low risk | No; low risk | No; low risk | No; low risk |
| Gerlier C, et al.[11] | Yes; low risk | Yes; low risk | Yes; low risk | low risk | low risk | Yes; low risk | low risk | Yes; low risk | Yes; low risk | Yes; low risk | Yes; low risk | low risk | Yes; low risk | Yes; low risk | low risk | low risk | No; low risk | No; low risk | No; low risk |
| Idil H, et al.[12] | Unclear | Yes; low risk | Yes; low risk | Unclear | unclear risk | Unclear | unclear | Unclear | Unclear | No; high risk | Yes; low risk | high risk | Unclear | Unclear | unclear risk | unclear | No; low risk | Unclear | Unclear |
| Jahn K, et al.[13] | Yes; low risk | Yes; low risk | Yes; low risk | low risk | high risk | Unclear | high risk | Unclear | Yes; low risk | Unclear | Yes; low risk | Unclear | No; high risk | Unclear | unclear risk | high risk | No; low risk | Unclear | Unclear |
| Kattah JC, et al.[14] | Yes; low risk | Yes; low risk | Yes; low risk | low risk | low risk | Yes; low risk | low risk | Yes; low risk | Yes; low risk | Yes; low risk | Yes; low risk | low risk | Yes; low risk | Yes; low risk | low risk | low risk | No; low risk | No; low risk | No; low risk |
| Kattah JC, et al.[15] | No; high risk | Yes; low risk | Yes; low risk | high risk | low risk | Yes; low risk | low risk | Yes; low risk | Yes; low risk | Yes; low risk | Yes; low risk | low risk | Yes; low risk | Yes; low risk | low risk | low risk | Unclear | No; low risk | No; low risk |
| Kerber KA, et al.[16] | Yes; low risk | Yes; low risk | Yes; low risk | low risk | high risk | Yes; low risk | high risk | Yes; low risk | Yes; low risk | Yes; low risk | Yes; low risk | low risk | Yes; low risk | Yes; low risk | unclear risk | unclear | Unclear | No; low risk | No; low risk |
| Kerber KA, et al.[17] | No; high risk | Yes; low risk | Yes; low risk | high risk | unclear risk | Unclear | unclear | Unclear | Unclear | Unclear | Yes; low risk | Unclear | Unclear | Unclear | unclear risk | unclear | Unclear | Unclear | No; low risk |
| Kim AS, et al.[18] | Yes; low risk | Yes; low risk | Yes; low risk | low risk | unclear risk | Unclear | unclear | Unclear | Unclear | No; high risk | Yes; low risk | high risk | Unclear | Unclear | unclear risk | unclear | Unclear | Unclear | Unclear |
| Kim MB, et al.[19] | Yes; low risk | Yes; low risk | Yes; low risk | low risk | low risk | No; high risk | high risk | Yes; low risk | No; high risk | Yes; low risk | No; high risk | high risk | Unclear | Yes; low risk | unclear risk | unclear | No; low risk | Unclear | No; low risk |
| Kogej M, et al.[20] | Yes; low risk | Yes; low risk | Yes; low risk | low risk | unclear risk | Unclear | unclear | Unclear | Unclear | Unclear | Yes; low risk | Unclear | Unclear | Unclear | unclear risk | unclear | Unclear | Unclear | Unclear |
| Korda A, et al.[21] | Yes; low risk | Yes; low risk | Yes; low risk | low risk | low risk | Yes; low risk | low risk | Yes; low risk | Yes; low risk | Yes; low risk | Yes; low risk | low risk | Yes; low risk | Yes; low risk | low risk | low risk | No; low risk | No; low risk | No; low risk |
| Korda A, et al.[22] | Yes; low risk | Yes; low risk | Yes; low risk | low risk | low risk | Yes; low risk | low risk | Yes; low risk | Yes; low risk | Yes; low risk | Yes; low risk | low risk | Yes; low risk | Yes; low risk | low risk | low risk | No; low risk | No; low risk | No; low risk |
| Lam JMY, et al.[23] | Yes; low risk | Yes; low risk | Yes; low risk | low risk | unclear risk | Unclear | unclear | Unclear | Unclear | Unclear | Yes; low risk | Unclear | Unclear | Unclear | unclear risk | unclear | Unclear | Unclear | Unclear |
| Lemos J, et al.[24] | Yes; low risk | Yes; low risk | Yes; low risk | low risk | low risk | Yes; low risk | low risk | Yes; low risk | Yes; low risk | Yes; low risk | Yes; low risk | low risk | Yes; low risk | Yes; low risk | unclear risk | unclear | No; low risk | No; low risk | No; low risk |
| Ljunggren M, et al.[25] | Yes; low risk | Yes; low risk | Yes; low risk | low risk | unclear risk | Unclear | unclear | Unclear | Unclear | Unclear | Yes; low risk | Unclear | Unclear | Unclear | unclear risk | unclear | No; low risk | Unclear | Unclear |
| Machner B, et al.[26] | Yes; low risk | Yes; low risk | Yes; low risk | low risk | low risk | Yes; low risk | low risk | Yes; low risk | Yes; low risk | Yes; low risk | Yes; low risk | low risk | Yes; low risk | Yes; low risk | unclear risk | unclear | No; low risk | No; low risk | No; low risk |
| Mantokoudis G, et [27]al. | Yes; low risk | Yes; low risk | Yes; low risk | low risk | low risk | Yes; low risk | low risk | Yes; low risk | Yes; low risk | Yes; low risk | Yes; low risk | low risk | Yes; low risk | Yes; low risk | low risk | low risk | No; low risk | No; low risk | No; low risk |
| Mantokoudis G, et al.[28] | Yes; low risk | Yes; low risk | Yes; low risk | low risk | low risk | Yes; low risk | low risk | Yes; low risk | Yes; low risk | Yes; low risk | Yes; low risk | low risk | Yes; low risk | Yes; low risk | low risk | low risk | No; low risk | No; low risk | No; low risk |
| Màrmol-Szombathy I, et al.[29] | No; high risk | Yes; low risk | Yes; low risk | high risk | unclear risk | Unclear | unclear | Unclear | Unclear | Unclear | Yes; low risk | Unclear | Unclear | Unclear | unclear risk | unclear | Unclear | Unclear | Unclear |
| Michailidou E, et al.[30] | Yes; low risk | Yes; low risk | Yes; low risk | low risk | low risk | Yes; low risk | low risk | Yes; low risk | Yes; low risk | Yes; low risk | Yes; low risk | low risk | Yes; low risk | Yes; low risk | low risk | low risk | No; low risk | No; low risk | No; low risk |
| Morrison M, et al.[31] | Yes; low risk | Yes; low risk | Yes; low risk | low risk | low risk | Yes; low risk | low risk | Yes; low risk | Yes; low risk | Yes; low risk | Yes; low risk | low risk | Yes; low risk | Yes; low risk | low risk | low risk | No; low risk | No; low risk | No; low risk |
| Navi BB, et al.[32] | Yes; low risk | Yes; low risk | Yes; low risk | low risk | unclear risk | Unclear | unclear | Unclear | Unclear | Unclear | Yes; low risk | Unclear | Unclear | Yes; low risk | unclear risk | unclear | Unclear | Unclear | Unclear |
| Newman-Toker DE, et al.[33] | Yes; low risk | Yes; low risk | Yes; low risk | low risk | unclear risk | Unclear | unclear | Unclear | No; high risk | No; high risk | Yes; low risk | high risk | Unclear | Yes; low risk | unclear risk | unclear | Unclear | Unclear | Unclear |
| Newman-Toker DE, et al.[34] | Yes; low risk | Yes; low risk | Yes; low risk | low risk | low risk | Yes; low risk | low risk | Yes; low risk | Yes; low risk | Yes; low risk | Yes; low risk | low risk | Yes; low risk | Yes; low risk | low risk | low risk | No; low risk | No; low risk | No; low risk |
| Nham B, et al.[35] | Yes; low risk | Yes; low risk | Yes; low risk | low risk | low risk | Yes; low risk | low risk | Yes; low risk | No; high risk | No; high risk | Yes; low risk | high risk | Yes; low risk | Yes; low risk | unclear risk | unclear | No; low risk | No; low risk | No; low risk |
| Nham B, et al.[36] | Yes; low risk | Yes; low risk | Yes; low risk | low risk | low risk | Yes; low risk | low risk | Yes; low risk | No; high risk | No; high risk | Yes; low risk | high risk | Yes; low risk | Yes; low risk | low risk | low risk | No; low risk | No; low risk | No; low risk |
| Ota K, et al.[37] | Yes; low risk | Yes; low risk | Yes; low risk | low risk | unclear risk | Unclear | unclear | Unclear | Unclear | Unclear | Yes; low risk | Unclear | Unclear | Unclear | unclear risk | unclear | Unclear | Unclear | Unclear |
| Royl G, et al.[38] | No; high risk | Yes; low risk | Yes; low risk | high risk | high risk | Yes; low risk | high risk | Yes; low risk | Unclear | Unclear | Yes; low risk | Unclear | Yes; low risk | Unclear | unclear risk | unclear | Unclear | Unclear | Unclear |
| Sandlund MG, et al.[39] | Yes; low risk | Yes; low risk | Yes; low risk | low risk | unclear risk | Unclear | unclear | Unclear | Unclear | Unclear | Unclear | Unclear | Unclear | Unclear | unclear risk | unclear | No; low risk | Unclear | Unclear |
| Sankalia D, et al.[40] | Yes; low risk | Yes; low risk | Yes; low risk | low risk | low risk | Yes; low risk | low risk | Yes; low risk | Yes; low risk | Yes; low risk | Yes; low risk | low risk | Yes; low risk | Yes; low risk | low risk | low risk | Unclear | No; low risk | No; low risk |
| Siepmann T, et al.[41] | Yes; low risk | Yes; low risk | Yes; low risk | low risk | unclear risk | Yes; low risk | unclear | Yes; low risk | Yes; low risk | Yes; low risk | Yes; low risk | low risk | Yes; low risk | Yes; low risk | low risk | low risk | No; low risk | No; low risk | No; low risk |
| Thomas JO, et al.[42] | Yes; low risk | Yes; low risk | Yes; low risk | low risk | low risk | Yes; low risk | low risk | Yes; low risk | Yes; low risk | Yes; low risk | Yes; low risk | low risk | Yes; low risk | Yes; low risk | low risk | low risk | No; low risk | No; low risk | No; low risk |
| Tong D, et al.[43] | No; high risk | Yes; low risk | Yes; low risk | high risk | unclear risk | Unclear | unclear | Unclear | Yes; low risk | Yes; low risk | Yes; low risk | Unclear | Unclear | Yes; low risk | unclear risk | unclear | Unclear | Unclear | No; low risk |
| von Werdt M, et al.[44] | Yes; low risk | Yes; low risk | Yes; low risk | low risk | low risk | Yes; low risk | low risk | Yes; low risk | Yes; low risk | Yes; low risk | Yes; low risk | low risk | Yes; low risk | Yes; low risk | low risk | low risk | No; low risk | No; low risk | No; low risk |
| Yebra González L, et al.[45] | Yes; low risk | Yes; low risk | Yes; low risk | low risk | high risk | Unclear | high risk | Unclear | Yes; low risk | Unclear | Yes; low risk | Unclear | No; high risk | Unclear | unclear risk | high risk | No; low risk | Unclear | Unclear |

References

1.	Carmona S, Martínez C, Zalazar G et al. (2016) The Diagnostic Accuracy of Truncal Ataxia and HINTS as Cardinal Signs for Acute Vestibular Syndrome. Frontiers in Neurology 7:125. https://doi.org/10.3389/fneur.2016.00125

2.	Casani AP, Dallan I, Cerchiai N et al. (2013) Cerebellar infarctions mimicking acute peripheral vertigo: how to avoid misdiagnosis? Otolaryngol Head Neck Surg 148:475–481. https://doi.org/10.1177/0194599812472614

3.	Chang Y-S, Tsai M-J, Hsieh C-Y et al. (2024) Characteristics and risk of stroke in emergency department patients with acute dizziness. Heliyon 10:e30953. https://doi.org/10.1016/j.heliyon.2024.e30953

4.	Chen L, Lee W, Chambers BR et al. (2011) Diagnostic accuracy of acute vestibular syndrome at the bedside in a stroke unit. Journal of neurology 258:855–861. https://doi.org/10.1007/s00415-010-5853-4

5.	Cheung CSK, Mak PSK, Manley KV et al. (2010) Predictors of important neurological causes of dizziness among patients presenting to the emergency department. Emerg Med J 27:517–521. https://doi.org/10.1136/emj.2009.078014

6.	Choi J-H, Park M-G, Choi SY et al. (2017) Acute Transient Vestibular Syndrome: Prevalence of Stroke and Efficacy of Bedside Evaluation. Stroke 48:556–562. https://doi.org/10.1161/STROKEAHA.116.015507

7.	Comolli L, Korda A, Zamaro E et al. (2023) Vestibular syndromes, diagnosis and diagnostic errors in patients with dizziness presenting to the emergency department: a cross-sectional study. BMJ Open 13:e064057. https://doi.org/10.1136/bmjopen-2022-064057

8.	Dmitriew C, Regis A, Bodunde O et al. (2021) Diagnostic Accuracy of the HINTS Exam in an Emergency Department: A Retrospective Chart Review. Acad Emerg Med 28:387–393. https://doi.org/10.1111/acem.14171

9.	Doijiri R, Uno H, Miyashita K et al. (2016) How Commonly Is Stroke Found in Patients with Isolated Vertigo or Dizziness Attack? J Stroke Cerebrovasc Dis 25:2549–2552. https://doi.org/10.1016/j.jstrokecerebrovasdis.2016.06.038

10.	Gerlier C, Fels A, Vitaux H et al. (2023) Effectiveness and reliability of the four-step STANDING algorithm performed by interns and senior emergency physicians for predicting central causes of vertigo. Acad Emerg Med 30:487–500. https://doi.org/10.1111/acem.14659

11.	Gerlier C, Hoarau M, Fels A et al. (2021) Differentiating central from peripheral causes of acute vertigo in an emergency setting with the HINTS, STANDING, and ABCD2 tests: A diagnostic cohort study. Acad Emerg Med 28:1368–1378. https://doi.org/10.1111/acem.14337

12.	Idil H, Ozbay Yenice G, Kilic TY et al. (2020) The Incidence of Central Neurological Disorders Among Patients With Isolated Dizziness and the Diagnostic Yield of Neuroimaging Studies. The neurologist 25:85–88. https://doi.org/10.1097/NRL.0000000000000282

13.	Jahn K, Kreuzpointner A, Pfefferkorn T et al. (2020) Telling friend from foe in emergency vertigo and dizziness: does season and daytime of presentation help in the differential diagnosis? Journal of neurology 267:118–125. https://doi.org/10.1007/s00415-020-10019-x

14.	Kattah JC, Badihian S, Pula JH et al. (2020) Ocular lateral deviation with brief removal of visual fixation differentiates central from peripheral vestibular syndrome. Journal of neurology 267:3763–3772. https://doi.org/10.1007/s00415-020-10100-5

15.	Kattah JC, Martinez C, Zalazar G et al. (2022) Role of incubitus truncal ataxia, and equivalent standing grade 3 ataxia in the diagnosis of central acute vestibular syndrome. Journal of the Neurological Sciences 441:120374. https://doi.org/10.1016/j.jns.2022.120374

16.	Kerber KA, Meurer WJ, Brown DL et al. (2015) Stroke risk stratification in acute dizziness presentations: A prospective imaging-based study. Neurology 85:1869–1878. https://doi.org/10.1212/WNL.0000000000002141

17.	Kerber KA, Meurer WJ, West BT et al. (2008) Dizziness presentations in U.S. emergency departments, 1995-2004. Acad Emerg Med 15:744–750. https://doi.org/10.1111/j.1553-2712.2008.00189.x

18.	Kim AS, Sidney S, Klingman JG et al. (2012) Practice variation in neuroimaging to evaluate dizziness in the ED. Am J Emerg Med 30:665–672. https://doi.org/10.1016/j.ajem.2011.02.038

19.	Kim M-B, Boo SH, Ban JH (2013) Nystagmus-based approach to vertebrobasilar stroke presenting as vertigo without initial neurologic signs. Eur Neurol 70:322–328. https://doi.org/10.1159/000353285

20.	Kogej M, Scherzberg J, Schacher S et al. (2024) Clinical use of the manchester triage system in patients with dizziness - An observational study in the emergency department. Int Emerg Nurs 73:101403. https://doi.org/10.1016/j.ienj.2023.101403

21.	Korda A, Wimmer W, Wyss T et al. (2022) Artificial intelligence for early stroke diagnosis in acute vestibular syndrome. Frontiers in Neurology 13:919777. https://doi.org/10.3389/fneur.2022.919777

22.	Korda A, Zamaro E, Wagner F et al. (2022) Acute vestibular syndrome: is skew deviation a central sign? Journal of neurology 269:1396–1403. https://doi.org/10.1007/s00415-021-10692-6

23.	Lam JM, Siu WS, Lam TS et al. (2006) The Epidemiology of Patients with Dizziness in an Emergency Department. Hong Kong Journal of Emergency Medicine 13:133–139. https://doi.org/10.1177/102490790601300302

24.	Lemos J, Martins AI, Duque C et al. (2019) Positional Testing in Acute Vestibular Syndrome: a Transversal and Longitudinal Study. Otol Neurotol 40:e119-e129. https://doi.org/10.1097/MAO.0000000000002067

25.	Ljunggren M, Persson J, Salzer J (2018) Dizziness and the Acute Vestibular Syndrome at the Emergency Department: A Population-Based Descriptive Study. Eur Neurol 79:5–12. https://doi.org/10.1159/000481982

26.	Machner B, Erber K, Choi JH et al. (2021) Usability of the head impulse test in routine clinical practice in the emergency department to differentiate vestibular neuritis from stroke. European Journal of Neurology 28:1737–1744. https://doi.org/10.1111/ene.14707

27.	Mantokoudis G, Korda A, Zee DS et al. (2021) Bruns' nystagmus revisited: A sign of stroke in patients with the acute vestibular syndrome. European Journal of Neurology 28:2971–2979. https://doi.org/10.1111/ene.14997

28.	Mantokoudis G, Wyss T, Zamaro E et al. (2021) Stroke Prediction Based on the Spontaneous Nystagmus Suppression Test in Dizzy Patients: A Diagnostic Accuracy Study. Neurology 97:e42-e51. https://doi.org/10.1212/WNL.0000000000012176

29.	Mármol-Szombathy I, Domínguez-Durán E, Calero-Ramos L et al. (2018) Identification of dizzy patients who will develop an acute cerebrovascular syndrome: a descriptive study among emergency department patients. Eur Arch Otorhinolaryngol 275:1709–1713. https://doi.org/10.1007/s00405-018-4988-2

30.	Michailidou E, Korda A, Wyss T et al. (2024) The value of saccade metrics and VOR gain in detecting a vestibular stroke. Journal of vestibular research : equilibrium & orientation 34:49–61. https://doi.org/10.3233/VES-230083

31.	Morrison M, Korda A, Zamaro E et al. (2022) Paradigm shift in acute dizziness: is caloric testing obsolete? Journal of neurology 269:853–860. https://doi.org/10.1007/s00415-021-10667-7

32.	Navi BB, Kamel H, Shah MP et al. (2012) Rate and predictors of serious neurologic causes of dizziness in the emergency department. Mayo Clin Proc 87:1080–1088. https://doi.org/10.1016/j.mayocp.2012.05.023

33.	Newman-Toker DE, Hsieh Y-H, Camargo CA et al. (2008) Spectrum of dizziness visits to US emergency departments: cross-sectional analysis from a nationally representative sample. Mayo Clin Proc 83:765–775. https://doi.org/10.4065/83.7.765

34.	Newman-Toker DE, Kerber KA, Hsieh Y-H et al. (2013) HINTS outperforms ABCD2 to screen for stroke in acute continuous vertigo and dizziness. Acad Emerg Med 20:986–996. https://doi.org/10.1111/acem.12223

35.	Nham B, Reid N, Bein K et al. (2022) Capturing vertigo in the emergency room: three tools to double the rate of diagnosis. Journal of neurology 269:294–306. https://doi.org/10.1007/s00415-021-10627-1

36.	Nham B, Wang C, Reid N et al. (2023) Modern vestibular tests can accurately separate stroke and vestibular neuritis. Journal of neurology 270:2031–2041. https://doi.org/10.1007/s00415-022-11473-5

37.	Ota K, Nishioka D, Katayama Y et al. (2023) Epidemiology of patients with dizziness over a 3-year period, requiring utilization of the emergency medical serviced system: A pre- and post-COVID pandemic comparative study using the population-based ORION registry. Journal of vestibular research : equilibrium & orientation 33:127–136. https://doi.org/10.3233/VES-220024

38.	Royl G, Ploner CJ, Leithner C (2011) Dizziness in the emergency room: diagnoses and misdiagnoses. Eur Neurol 66:256–263. https://doi.org/10.1159/000331046

39.	Sandlund MG, Diamant A, Granåsen G et al. (2019) Effectiveness of care in acute dizziness presentations. Eur Arch Otorhinolaryngol 276:2389–2396. https://doi.org/10.1007/s00405-019-05470-0

40.	Sankalia D, Kothari S, Phalgune DS (2021) Diagnosing Stroke in Acute Vertigo: Sensitivity and Specificity of HINTS Battery in Indian Population. Neurol India 69:97–101. https://doi.org/10.4103/0028-3886.310089

41.	Siepmann T, Gruener C, Simon E et al. (2021) Video-Oculography-Assisted Head Impulse Test and Caloric Testing for Detecting Stroke in Acute Vertigo Patients via Modified HINTS Plus. J Clin Med 10. https://doi.org/10.3390/jcm10194471

42.	Thomas JO, Sharobeam A, Venkat A et al. (2022) Video head impulse testing to differentiate vestibular neuritis from posterior circulation stroke in the emergency department: a prospective observational study. BMJ Neurology Open 4:e000284. https://doi.org/10.1136/bmjno-2022-000284

43.	Tong D, Chen X, Wang Y et al. (2020) Acute and episodic vestibular syndromes caused by ischemic stroke: predilection sites and risk factors. J Int Med Res 48:300060520918039. https://doi.org/10.1177/0300060520918039

44.	Werdt M von, Korda A, Zamaro E et al. (2024) The acute vestibular syndrome: prevalence of new hearing loss and its diagnostic value. Eur Arch Otorhinolaryngol 281:1781–1787. https://doi.org/10.1007/s00405-023-08296-z

45.	Yebra González L, González Márquez R, Rueda Marcos A et al. (2021) Protocolo de vértigo de origen dudoso (Unclear origin vertigo protocol). Acta Otorrinolaringol Esp (Engl Ed) 72:92–100. https://doi.org/10.1016/j.otorri.2020.02.012
